# Structure‐Tailoring Cerium Nanozymes with Self‐Cascade ROS Scavenging Catalysis Modulate the Microbiota‐Gut‐Joint Axis for Rheumatoid Arthritis Therapy

**DOI:** 10.1002/advs.202512281

**Published:** 2025-09-29

**Authors:** Ge Wang, Xueqing Zhang, Boyuan Zhu, Suyue Ding, Dong Yan, Jing Ma, Yafang Xiao, Yafu Wang, Tianjun Ni, Hua Zhang, Weisheng Guo

**Affiliations:** ^1^ School of Basic Medical Sciences Xinxiang Medical University School of Pharmacy Xinxiang Medical University The First Affiliated Hospital of Xinxiang Medical University Xinxiang 453007 P. R. China; ^2^ Key Laboratory of Green Chemical Media and Reactions Ministry of Education Henan Key Laboratory of Organic Functional Molecule and Drug Innovation School of Chemistry and Chemical Engineering Henan Normal University Xinxiang 453007 P. R. China; ^3^ Department of Minimally Invasive Interventional Radiology The Second Affiliated Hospital School of Biomedical Engineering Guangzhou Medical University Guangzhou 510260 P. R. China

**Keywords:** cerium nanozymes, gut‐joint axis, rheumatoid arthritis, self‐cascade

## Abstract

Rheumatoid arthritis (RA) is closely associated with intestinal microbiota dysbiosis, highlighting the therapeutic potential of targeting the microbiota‐gut‐joint axis. Current interventions often overlook the cascade nature of reactive oxygen species (ROS) generation in driving intestinal and systemic inflammation. Herein, a valence‐engineered CeO_X_‐based nanozyme with self‐cascade catalytic activity is developed, mimicking sequential oxidase‐superoxide dismutase‐peroxidase functions to enable continuous ROS scavenging while minimizing oxygen generation. By precisely tuning Ce^3+^/Ce^4+^ ratios from 0.27 to 0.93 through Au deposition (0.23 wt.%→5.2 wt.%), Dual functionality is achieved: 1) enhanced oxygen vacancy generation (71.4%) for efficient ROS scavenging via superoxide anion→hydrogen peroxide→hydroxide ion conversion, and 2) suppressed oxygen production to maintain the anaerobic microenvironment essential for gut microbiota. Encapsulating the nanozyme with sodium alginate (SA) to form Au/CeO_X_(0.93)@SA ensures resistance to gastric acid upon oral administration. In RA model rats, this strategy restored gut microbial balance, normalized short‐chain fatty acid profiles, and significantly attenuated joint inflammation and cartilage degradation. The therapeutic efficacy is further evidenced by reduced systemic pro‐inflammatory cytokine levels and improved intestinal barrier integrity. This study established a design paradigm for gut microenvironment‐adapted nanozymes, offering a dual‐action strategy for early RA intervention through synchronized ROS elimination and microbiota homeostasis restoration.

## Graphical abstract

A valence‐engineered CeOX nanozyme, with its Ce^3+^/Ce^4+^ ratio precisely controlled within the range of 0.27 to 0.93 through Au deposition, performs a self‐cascade oxidase‐superoxide dismutase‐peroxidase reaction, enabling continuous reactive oxygen species scavenging while minimizing oxygen generation. Oral sodium alginate‐microencapsulated CeOX nanozyme restores gut microbial homeostasis and exerts dual‐action for early rheumatoid arthritis intervention.

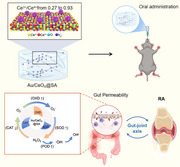

## 1 Introduction

Rheumatoid arthritis (RA) is a systemic autoimmune disease characterized by chronic inflammation and progressive joint destruction.^[^ ^1^ ^]^ Conventional treatments, such as oral disease‐modifying antirheumatic drugs and intra‐articular glucocorticoid injections, primarily target overt pathological changes to alleviate joint swelling and pain.^[^ ^2^, ^3^ ^]^ However, these approaches often yield suboptimal outcomes and are associated with significant side effects, including immune suppression, gastrointestinal complications, and inadequate long‐term disease control.^[^ ^4^ ^]^ Interventions targeting the preclinical phase of RA hold promise but remain limited. Emerging evidence highlights the critical role of gut‐organ axes in systemic inflammatory diseases, exemplified by the gut‐joint axis in RA,^[^ ^5^, ^6^ ^]^ the gut‐skin axis in psoriasis,^[^ ^7^ ^]^ and the gut‐brain axis in Alzheimer's disease.^[^ ^8^, ^9^ ^]^ In the context of RA, intestinal dysbiosis likely plays a crucial role in triggering the initial inflammatory cascade.^[^ ^6^, ^10^ ^]^ Once RA is established, interventions targeting dysbiosis may no longer effectively modify the course of RA. Therefore, modulating gut microbiota is an effective strategy for preventing the onset of RA.

In RA progression, a key pathogenic mechanism involves dysregulated gut microbiota that drive host cells to over‐produce superoxide anions (O_2_^•−^).^[^ ^11^ ^]^ As the primary precursor in reactive oxygen species (ROS) formation, O_2_^•−^ undergoes sequential conversion to hydrogen peroxide (H_2_O_2_), hydroxyl radicals (·OH), and hydroperoxyl radicals through a cascade reaction, thereby amplifying oxidative stress.^[^ ^12^, ^13^ ^]^ This cascading oxidative process continuously generates excessive ROS, jointly intensifies intestinal permeability (e.g., oxygen leakage), further aggravates gut microbiota dysbiosis, and fuels systemic inflammation, including RA progression.^[^ ^14^, ^15^ ^]^ Therefore, early intervention in RA should prioritize the stepwise elimination of excess ROS within the intestinal microenvironment, for example, sequentially scavenging O_2_^•−^ → H_2_O_2_ → •OH to modulate the microbiota‐gut‐joint axis.

Mixed‐valence metal‐based nanozymes (e.g., CeO_2_, Co_3_O_4_, V_4_C_3_) have emerged as versatile candidates for ROS regulation due to their tunable enzyme‐mimetic catalytic activities.^[^ ^16^, ^17^ ^]^ These nanomaterials exhibit oxidase (OXD)‐, superoxide dismutase (SOD)‐, peroxidase (POD)‐, glucose oxidase (GOx)‐, or catalase (CAT)‐like functions, with catalytic performance dictated by surface defect vacancies and metal valence states.^[^ ^18^, ^19^, ^20^ ^]^ Recent advances in valence engineering—such as lower‐valence metal doping, single‐atom incorporation, or active‐site modification—enable precise control over catalytic behavior. For instance, Ir‐anchored CeO_2_ nanoislands demonstrate GOx‐POD cascade activity, while Mn‐Ce hybrid nanocrystals exhibit synergistic SOD‐CAT functions.^[^ ^21^, ^22^ ^]^ Such self‐cascade single‐enzyme systems enhance reaction efficiency by optimizing intermediate transport and minimizing enzyme interference, offering potential for sustained ROS elimination in biological systems. However, when single‐enzyme ROS quenchers are deployed to modulate the microbiota‐gut‐joint axis, they should both extinguish ROS cascades and maintain strict anaerobiosis to safeguard a healthy gut niche.

**Scheme 1:**
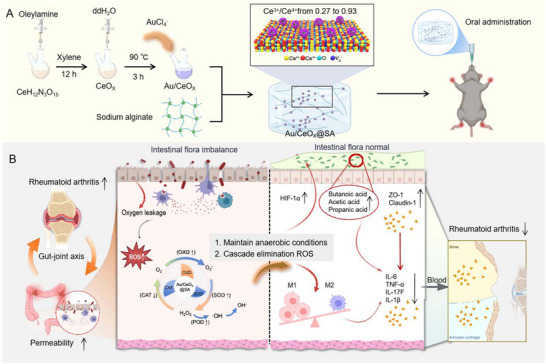
Schematic diagram showing the self‐cascade enzyme‐like activities of Au/CeO_X_@SA in regulating the microbiota‐gut‐joint axis. A) The preparation and structural features of Au/CeO_X_@SA; B) Their cascade enzyme‐like activities, along with their anti‐inflammatory effects on rheumatoid arthritis through the regulation of the microbiota‐gut‐joint axis.

Building on these insights, we engineered a CeO_X_ based single‐enzyme platform with integrated OXD‐SOD‐POD cascade activity (**Scheme** **1**). By employing noble metal deposition, we systematically elevated the Ce^3+^/Ce^4+^ ratio from 0.27 to 0.93, correlating Au doping concentration with oxygen vacancy (*V_O_*) generation. This valence engineering strategy facilitated electron transfer from Au to CeO_X_ surfaces, yielding 71.4% oxygen vacancies and reduced Ce valence states. Crucially, the modified system lowered the free energy barrier for O_2_^•−^‐H_2_O_2_‐OH^−^ cascade reactions while suppressing CAT‐like activity (due to diminished Ce^4+^), thereby minimizing oxygen release and preserving anaerobic gut microbiota. To ensure gastrointestinal stability, we encapsulated the optimized Au/CeO_X_(0.93) nanozyme within a sodium alginate (SA) matrix, forming an acid‐resistant Au/CeO_X_(0.93)@SA composite. In RA model rats, this formulation effectively restored gut microbial homeostasis, normalized short‐chain fatty acid metabolism, and significantly attenuated disease progression, demonstrating its potential for early RA intervention.

## 2 Results and Discussion

### 2.1 Preparation and Characterization of Au/CeO_X_

In this study, CeO_X_ was employed as a reducing agent to facilitate the spontaneous growth of Au. To optimize the Ce^3+^/Ce^4+^ ratio, a valence state regulation strategy was applied by systematically doping CeO_X_ with Au at varying concentrations (0–10 wt.%). 5 CeO_X_ nanozymes with distinct Ce^3+^/Ce^4+^ ratios were synthesized and designated as CeO_X_(0.27), Au/CeO_X_(0.49), Au/CeO_X_(0.50), Au/CeO_X_(0.77), and Au/CeO_X_(0.93) (the number represent the Ce^3+^/Ce^4+^ ratio). X‐ray diffraction (XRD) analysis (Figure S1, Supporting Information) confirmed the successful preparation of Au/CeO_X_ nanoclusters. The diffraction peaks of CeO_X_ at 28.55, 33.08, 47.48, 56.34, and 59.09° correspond to the (111), (200), (220), (311), and (222) crystal planes of the cubic fluorite structure, respectively. The Au diffraction peaks at 38.18, 44.39, 64.57, and 77.54° match the (111), (200), (220), and (311) crystal planes of Au nanoparticles, consistent with the standard card (04‐0784).

**Figure 1:**
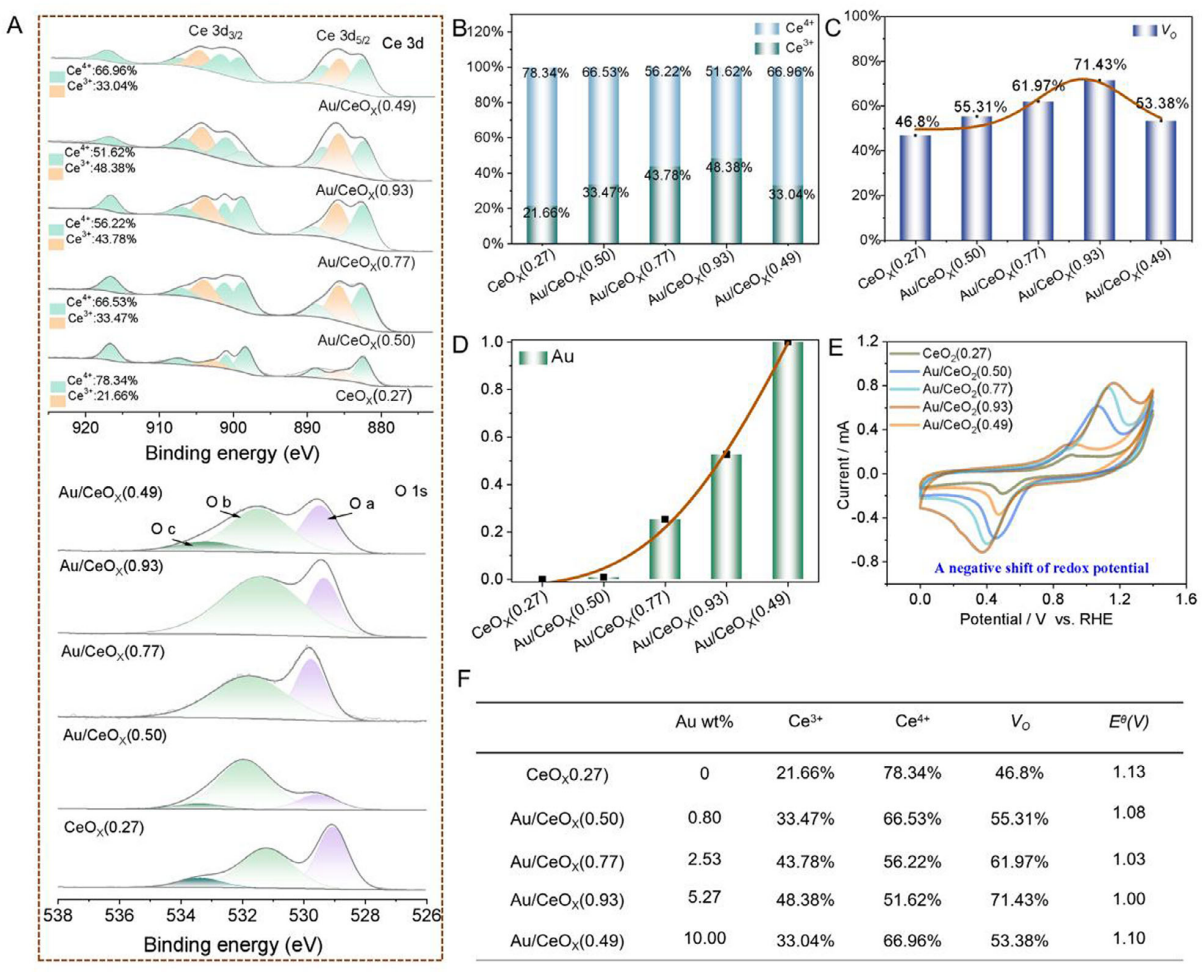
Chemical and electronic structures analysis of CeO_X_(0.27), Au/CeO_X_(0.50), Au/CeO_X_(0.77), Au/CeO_X_(0.93) and Au/CeO_X_(0.49). A) XPS spectra of Ce 3d (Ce 3d_3/2_ and Ce 3d_5/2_) and O 1s (O_a_: lattice oxygen; O_b_: oxygen vacancy; O_c_: adsorbed oxygen); B) The valence ratio analysis for Ce^3+^ and Ce^4+^; C) Oxygen vacancy analysis of five kinds of CeO_X_; D) The amount of Au deposition in five kinds of CeO_X_; E) Cyclic voltammetry curve of five kinds of CeO_X_; F) The table for comparison of five kinds of CeO_X_ with different valence ratio.

X‐ray photoelectron spectra (XPS) measurements were conducted to analyze electronic information of Ce and O. The results in **Figures** **1**A and S2 (Supporting Information) show that Au doping has a significant influence on the Ce^3+^/Ce^4+^ ratio in CeO_X_. Compared with the pristine Ce^3+^/Ce^4+^ ratio in CeO_X_(0.27) (0 wt.% Au), all Au‐containing samples exhibit a higher Ce^3+^/Ce^4+^ ratio, ascribed to electron transfer from Au to CeO_X_ that stabilizes Ce^3+^. With optimal Au doping (5.27 wt. %), this ratio increases gradually to its maximum value of 0.93. However, further increasing the Au doping to 10 wt.% results in a decrease of the Ce^3+^/Ce^4+^ ratio to 0.49 (Figure 1B,D). Additionally, the Ce^3+^ content in CeO_X_ increases from 21.6% to 48.38% upon Au deposition, while the oxygen vacancies (*V_O_*) concentration rises from 48.32% to 71.43% (Figure 1C,D). XPS reveals that Au exists as Au⁰, Au^+^ and Au^3+^ (Figure S3, Supporting Information). The 0.93 Au/CeO_X_ sample (5.27 wt.% Au), which displays the highest Ce^3+^/Ce^4+^ ratio, contains predominantly cationic Au (Au^+^ and Au^3+^) (Figure S3, Table S1, Supporting Information), markedly exceeding the cationic Au content in Au/CeO_X_(0.50) (0.80 wt.% Au) and Au/CeO_X_(0.77) (2.53 wt.% Au). Strikingly, further increasing the Au loading to 10.0 wt.% (Au/CeO_X_(0.49)) lowers the Ce^3+^/Ce^4+^ ratio from 0.93 to 0.49, because excessive Au agglomerates into large nanoparticles that weaken Au–Ce interactions and favor Au^0^ and Au^+^ formation. High‐resolution transmission electron microscopy (HRTEM) images (Figure S4, Supporting Information) reveal that the density and size of Au nanoparticles increase with higher Au deposition amounts. Consequently, an optimal Au loading secures highly dispersed Au clusters, maximizes Au‐CeO_X_ electron transfer, and yields the highest Ce^3^⁺ content in Au/CeO_X_(0.93).

We also employed Bader charge analysis to investigate the effect of doping on charge transfer. As shown in Figure S5 (Supporting Information), in Au/CeO_X_(0.93) (Au doping: 5.27 wt.%), each Au atom donates 0.12e to CeO_X_ (Figure S5A, Supporting Information), whereas in Au/CeO_X_(0.49) (Au doping: 10.0 wt.%), the donation is only 0.02e per Au atom (Figure S5B, Supporting Information). This indicates that excessive Au loading suppresses electron transfer, a trend corroborated by the XPS results (Figure S3, Supporting Information). Consequently, the Ce^3+^/Ce^4+^ ratio initially increases with Au loading, which can be ascribed to the asymmetric Ce‐O‐Au configuration that strengthens metal‐support interaction, and promotes electron transfer.^[^ ^23^, ^24^ ^]^ Beyond a certain point, however, the Ce^3^⁺/Ce⁴⁺ ratio declines because overly large Au clusters reduce the interfacial Au‐O‐Ce area and weaken the metal‐support interaction, thereby hindering charge transfer.^[^ ^25^, ^26^ ^]^

Considering that Ce^3+^/Ce^4+^ ratio in CeO_X_ affects its redox reactivity with ROS, the redox potentials of the 5 nanozymes were determined by measuring their cyclic voltammetry measurements (Figure 1E). The standard redox potential of CeO_X_(0.27) is 1.13 V, while that of Au/CeO_X_(0.93) is 1.00 V. These results indicate that the lower redox potential of Au/CeO_X_(0.93) exhibits the most outstanding potential for removal or retention of ROS. The corresponding Ce^3+^ and Ce^4+^ contents are summarized in Figure 1F.

### 2.2 Self‐Cascade Enzyme‐Like Property of Au/CeO_X_

**Figure 2:**
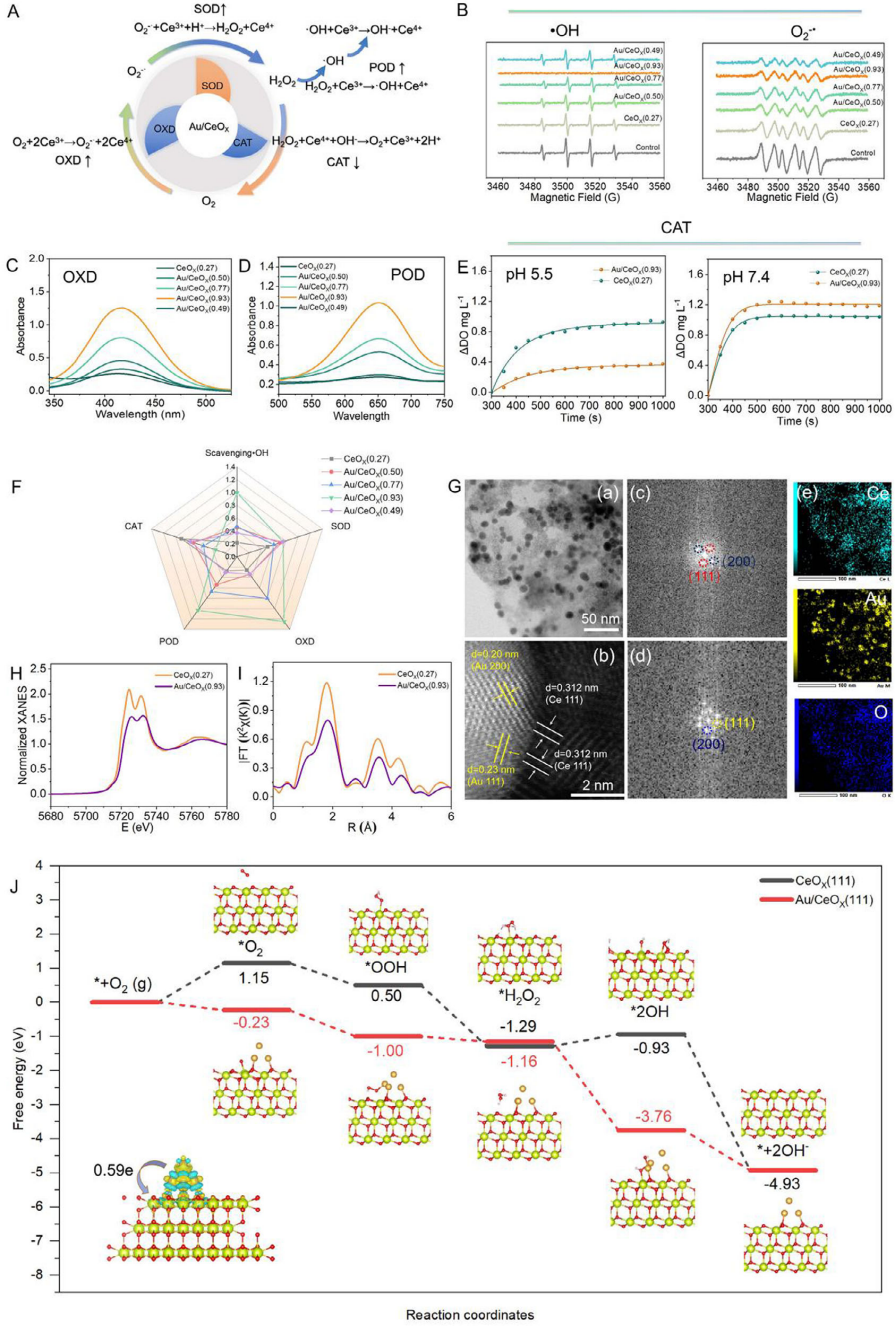
A) Schematic illustration of enzyme‐like activities of Au/CeO_X_; B) ESR spectra of •OH and O_2_^−•^ trapped by DMPO after being treated with five kinds of CeO_X_ respectively; C) UV−vis absorption spectra at 426 nm: OXD‐like activity detection with OPD probe (OPD: 27 mM, sample: 5 µg mL^−1^, five kinds of CeO_X_ with varying valence ratios); D) UV−vis spectra at 652 nm: POD‐like activity of five CeO_X_ (5 µg mL^−1^) with different valence ratios, detected by TMB probe (TMB: 1 mM, H_2_O_2_: 10 mM); E) Generation of dissolved oxygen from the decomposition of H_2_O_2_ (10 mM) with CeO_X_(0.27) or Au/CeO_X_(0.93) treatments in pH 5.5 and pH 7.4 buffer (Sample:20 µg mL^−1^); F) Radar map of biocatalytic ROS‐elimination properties based on SOD, OXD, POD and CAT‐like activities; G) G‐a: HRTEM image of Au/CeO_X_(0.93); G‐b: HAADF‐STEM image of Au/CeO_X_(0.93); G‐c and G‐d: Selected‐area FFT patterns of Au/CeO_X_(0.93) marking (G‐c)Ce (111)/(200), and (G‐d)Au (111)/(200), respectively; G‐e: Element mapping displaying the distribution of Ce, Au and O for Au/CeO_X_(0.93); H) Normalized XANES spectra at the Ce K‐edge of CeO_X_(0.27) and Au/CeO_X_(0.93); I) Ce L3‐edge Fourier transform EXAFS spectra of CeO_X_(0.27) and Au/CeO_X_(0.93); J) Free energy profiles for ROS‐elimination properties on the CeO_X_(0.27) and Au/CeO_X_(0.93) based on Ce(111) crystal plane of. Insert: electronic transfer at the Ce‐Au interface.

A series of self‐cascading nanozyme activities was evaluated according to the schematic illustration of enzyme‐like activities in **Figure** **2**A. O‐phenylenediamine (OPD) was used as the substrate to assess the OXD‐like catalytic activity (O_2_+2Ce^3+^→O_2_^•−^+2Ce^4+^) via ultraviolet‐visible (UV) spectroscopy. Meanwhile, the SOD‐like catalytic activity was evaluated by electron spin resonance (ESR) spectroscopy through the scavenging of O_2_^•−^ and •OH (•OH+Ce^3+^→OH^−^+Ce^4+^, O_2_^•−^+Ce^3+^+H^+^→H_2_O_2_+Ce^4+^). As shown in Figure 2B,C, based on the deposition of Au on the surface of CeO_X_, which increases the ratio of Ce^3+^ on the CeO_X_ surface, a higher OXD‐like and SOD‐like activity is exhibited. The highest OXD‐like and SOD‐like activities exhibited by Au/CeO_X_(0.93) can be attributed to the highest increased ratio of Ce^3+^ on the CeO_X_ surface, facilitating the manifestation of OXD‐like and SOD‐like activities. Furthermore, the most excellent POD‐like activity of Au/CeO_X_(0.93) was also confirmed when 3,3′,5,5′‐Tetramethylbenzidine (TMB) was used as the substrate for colorimetric detection (Figure 2D). At this point, the self‐cascading OXD‐SOD(O_2_^•−^)‐POD enzyme activities have been confirmed for continuous removing ROS.

Additionally, the CAT‐like activity, which decomposes H_2_O_2_ into H_2_O and O_2_ (H_2_O_2_+Ce^4+^+OH^−^→O_2_+Ce^3+^+2H^+^), was evaluated by monitoring dissolved oxygen levels. Minimizing oxygen generation is essential, as the nanozymes are designed to remove ROS via cascade catalysis while maintaining the anaerobic environment required by the predominantly anaerobic intestinal microbiota. As the results shown in Figure 2E, under neutral conditions, Au/CeO_X_(0.93) produced slightly more O_2_ than CeO_X_(0.27). Interestingly, under weakly acidic conditions, the CAT enzyme activity of Au/CeO_X_(0.93) was significantly reduced, indicating that the potential O_2_ production might be significantly lower compared to that of CeO_X_(0.27). The Au/CeO_X_(0.93) single‐enzyme system, while unable to completely prevent oxygen production during ROS elimination, has been optimized to maximize the environmental suitability for beneficial gut microbiota survival. This tailored enzymatic activity is attributed to the minimized proportion of Ce^4+^. The OXD‐, POD‐, CAT‐, SOD‐like properties are compared in Figure 2F based on changes in valence ratios.

### 2.3 Comparative Structural and Theoretical Analysis of Au/CeO_X_(0.93) and CeO_X_(0.27)

To understand the origin of the enzymatic activity of Au/CeO_X_(0.93), we selected two typical samples, Au/CeO_X_(0.93) and CeO_X_(0.27), for in‐depth comparative analysis. High‐angle annular dark‐field scanning transmission electron microscopy (HAADF‐STEM) images (Figure 2G) clearly reveal the lattice fringes corresponding to the (111) and (200) crystal planes of Au, as well as the (111) plane of CeO_X_. These images also demonstrate tight lattice intercalation at the Ce‐Au interface. Fast Fourier transform (FFT) patterns further confirm that the main crystal planes of Ce and Au are consistent with both XRD and HAADF‐STEM analyses. Energy‐dispersive spectroscopy (EDS) mappings indicate that Ce, O, and Au are uniformly distributed in the Au/CeO_X_(0.93) nanozymes, similar to other Au/CeO_X_ nanozymes (Figure S6, Supporting Information). We also collected infrared spectra to investigate the structural information. As depicted in Figure S7 (Supporting Information), the band at 520 cm^−1^ corresponds to the stretching vibration of the Ce‐O bond. Compared to CeO_X_(0.27), the enhanced and broadened absorption band at 3441 and 520 cm^−1^ in Au/CeO_X_(0.93) can be observed, suggesting that Au deposition alters the surface chemical environment of cerium oxide. The loading of Au induces lattice strain in cerium oxide, causing a shift in the Ce‐O stretching frequency.

To further elucidate the electronic structure and coordination environment Ce after Au deposition, X‐ray absorption near‐edge structure (XANES) and X‐ray absorption fine structure (EXAFS) experiments were conducted at Ce L3‐edge. As shown in Figure 2H, the XANES spectra of Au/CeO_X_(0.93) and CeO_X_(0.27) align with previously reported data.^[^ ^27^ ^]^ Compared to CeO_X_(0.27), the intensity of the white line in Au/CeO_X_(0.93) is significantly reduced, indicating a decrease in the average oxidation state of Ce after Au deposition, which is associated with the reduction of Ce^4+^. The EXAFS spectra (Figure 2I) reveal two prominent peaks at 1.8 and 3.6 Å for Au/CeO_X_(0.93), corresponding to the first (Ce‐O) and second (Ce‐Ce) coordination shells of Ce atoms, respectively. The positions of these two peaks in Au/CeO_X_(0.93) almost remains unchanged compared to CeO_X_(0.27), confirming that Au doping does not alter the fluorite structure of pristine CeO_X_. However, the amplitudes of these peaks, particularly the first shell, are notably diminished, indicating a reduction in the Ce‐O coordination number. This suggests that Au doping introduces lattice defects into CeO_X_.

To further elucidate the underlying catalytic mechanism of Au/CeO_X_(0.93) and CeO_X_(0.27), we conducted density functional theory (DFT) calculations using the Vienna Ab Initio Package within the generalized gradient approximation using the PBE formulation.^[^ ^28^, ^29^ ^]^ As illustrated in Figure 2J, we calculated the Gibbs energy for Au/CeO_X_(0.93) and CeO_X_(0.27). For Au/CeO_X_(0.93), the Gibbs energy of adsorption of O_2_ (−0.23 eV) is significantly negative than that of the CeO_X_(0.27) configuration (1.15 eV). Additionally, we computed the adsorption energies of O_2_ on both CeO_X_(0.27) and Au/CeO_X_(0.93). For CeO_X_(0.27), the value is−0.085 eV, whereas for Au/CeO_X_(0.93) it is−1.517 eV. Then, this process is followed by two consecutive exothermic steps via the ·OOH intermediate, leading to the formation of *H_2_O_2_, with respective energy changes of−1.00 and−1.16 eV. The presence of abundant oxygen vacancies and Ce^3+^ in Au/CeO_X_ (0.93) synergistically promotes the decomposition of H_2_O_2_, converting *OH to OH^−^ with an endothermic energy change of−3.76 eV. Conversely, CeO_X_(0.27) favors the desorption of H_2_O_2_, with an endothermic energy change of−0.93 eV. These findings indicate that the strong interface coupling between Au and CeO_X_ in Au/CeO_X_(0.93) facilitates efficient electron transfer from the Au surface to the Ce(111) surface, thereby reducing the activation energy required for these reactions and enhancing overall catalytic efficiency.

### 2.4 Assessing the Intracellular ROS Scavenging Ability of Au/CeO_X_(0.93) and CeO_X_(0.27)

**Figure 3:**
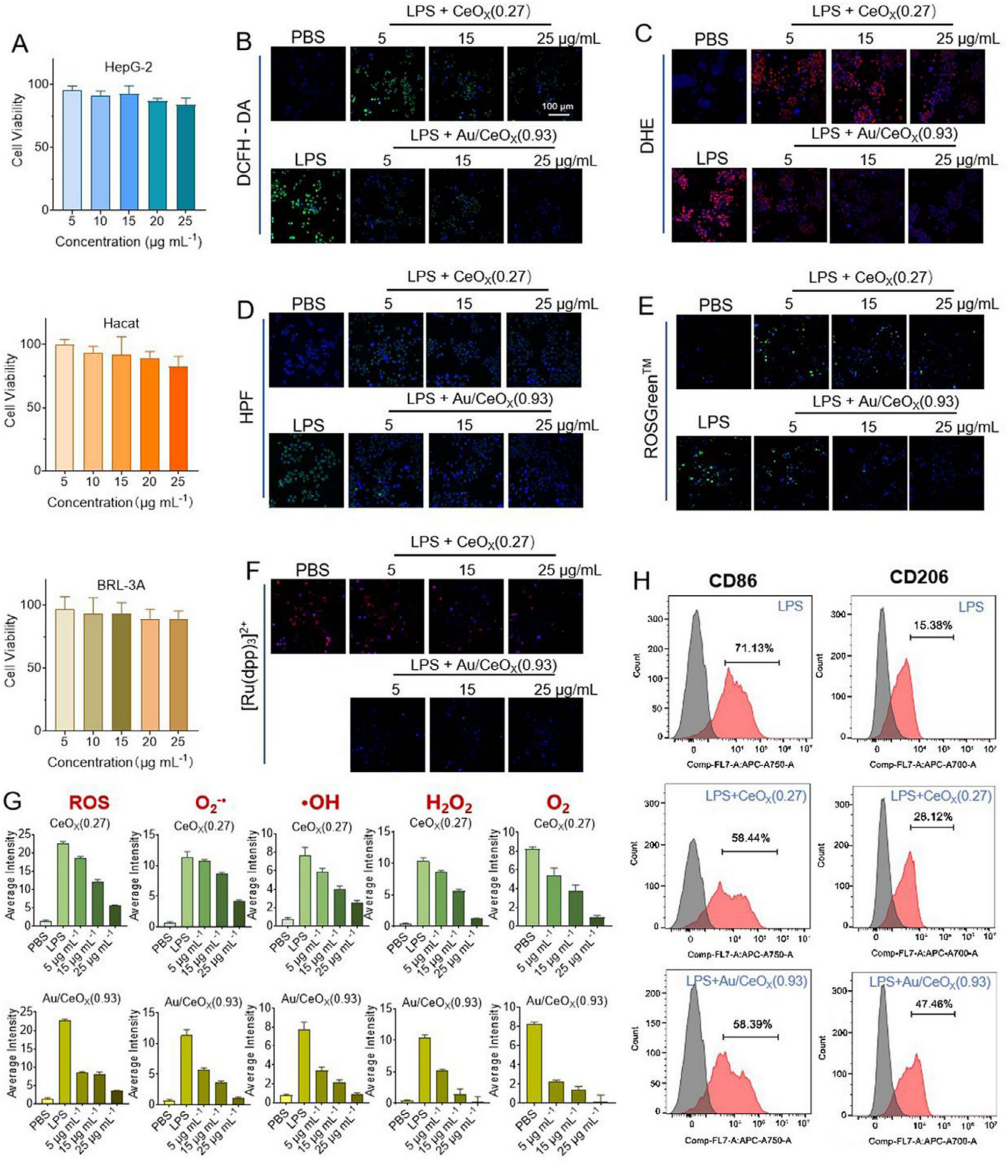
A) Cell viability of HepG2, HaCat and BRL‐3A, incubated with different concentrations of Au/CeO_X_(0.93) for 24 h. All data are presented as mean ± SD (*n* ≥ 3); Confocal laser scanning microscopy images of LPS inducing RAW264.2 cells under different concentration treatments of CeO_X_(0.27) and Au/CeO_X_(0.93) for B) detecting ROS levels when using DCFH‐DA probe, for C) detecting O_2_^−•^ levels when using DHE probe, for D) assaying •OH when using HPF probe, for E) detecting H_2_O_2_ levels when using ROSGreen^TM^ probe and for (E) detecting O_2_ levels when using [Ru(dpp)_3_]^2+^ probe. G) Fluorescence intensity analysis under different concentration treatments of CeO_X_(0.27) and Au/CeO_X_(0.93). All imaged cells are co‐incubated with DAPI (blue). All data are presented as mean ± SD (*n* ≥ 3); H) M1/M2 macrophage polarization analysis by flow cytometry.

To rigorously evaluate the biosafety of Au/CeO_X_(0.93), cytotoxicity assays were conducted using representative cell lines: HepG‐2 (human hepatocellular carcinoma), HaCaT (human keratinocyte), and BRL 3A (rat liver cell). As shown in **Figure** **3**A, within the concentration range of 5–25 µg mL^−1^, the cell viability of these three cell lines exceeded 80% compared to the control group without sample addition. Moreover, a hemolysis experiment is performed for a biosafety evaluation. Based on the hemolysis data, Au/CeO_X_(0.93) do not induce hemolysis (Figure S8, Supporting Information). These results demonstrate that CeO_X_ nanozymes exhibit negligible cytotoxicity within this concentration range and possess excellent biocompatibility, thereby providing a crucial cellular‐level safety basis for their potential applications in the biomedical field, such as the treatment of RA. To assess the ROS scavenging ability of CeO_X_ nanozymes, the levels of ROS, O_2_^•−^, •OH, and H_2_O_2_ were examined in the RAW 264.7 macrophage inflammation model induced by 100 ng mL^−1^ lipopolysaccharide (LPS). 2′,7′‐dichlorodihydrofluorescein diacetate (DCFH‐DA), dihydroethidium (DHE), 4‐Hydroxyphenyl fluorescein (HPF) and ROSGreen probes, respectively (Figures 3B–E; S9, Supporting Information). Au/CeO_X_(0.93), which possesses a higher Ce^3+^/Ce^4+^ ratio (0.93), exhibited significantly superior scavenging of ROS, O_2_^•−^, •OH, and H_2_O_2_ compared with samples having a lower Ce^3^⁺/Ce⁴⁺ ratio (0.27). This enhanced ability was concentration‐dependent. As anticipated, the consumed H_2_O_2_ was not converted to oxygen by catalase, instead, the [Ru(dpp)_3_]^2+^ probe revealed a decrease in oxygen level, most pronounced in the Au/CeO_X_(0.93) group (Figures 3F; S10, Supporting Information). Consequently, the catalytic cascade of Au/CeO_X_(0.93) proceeds via the OXD‐SOD‐POD sequence rather than the OXD‐SOD‐CAT pathway.

Given the close relationship between macrophage polarization (M1/M2 phenotypes) and intracellular ROS levels, we further examined the expression of CD86 (an M1‐specific marker) and CD206 (an M2‐specific marker) via flow cytometry to directly confirm the phenotypic switch of macrophages treated with Au/CeO_X_(0.93) and CeO_X_(0.27). Flow cytometry results (Figure 3H) showed that both Au/CeO_X_(0.93) and CeO_X_(0.27) inhibited LPS‐induced CD86 expression. The Au/CeO_X_(0.93) group exhibited a lower percentage of CD86‐positive cells (58.39%) compared to the CeO_X_(0.27) group, indicating superior suppression of the M1 phenotype. For CD206 expression, the CeO_X_(0.27) group (28.12%) showed no significant difference from the negative control group (15.36%), whereas the Au/CeO_X_(0.93) group demonstrated a substantial increase (47.46%), suggesting a pronounced shift toward the M2 phenotype. Thus, Au/CeO_X_(0.93) not only enhanced antioxidant properties through ROS scavenging but also exerted a synergistic effect on inflammatory inhibition, ensuring a more effective and stable transition from the M1 to the M2 phenotype.

### 2.5 Evaluating Gut Microbiota Restoration in RA via Oral Administration of Encapsulated Au/CeO_X_(0.93)@SA

**Figure 4:**
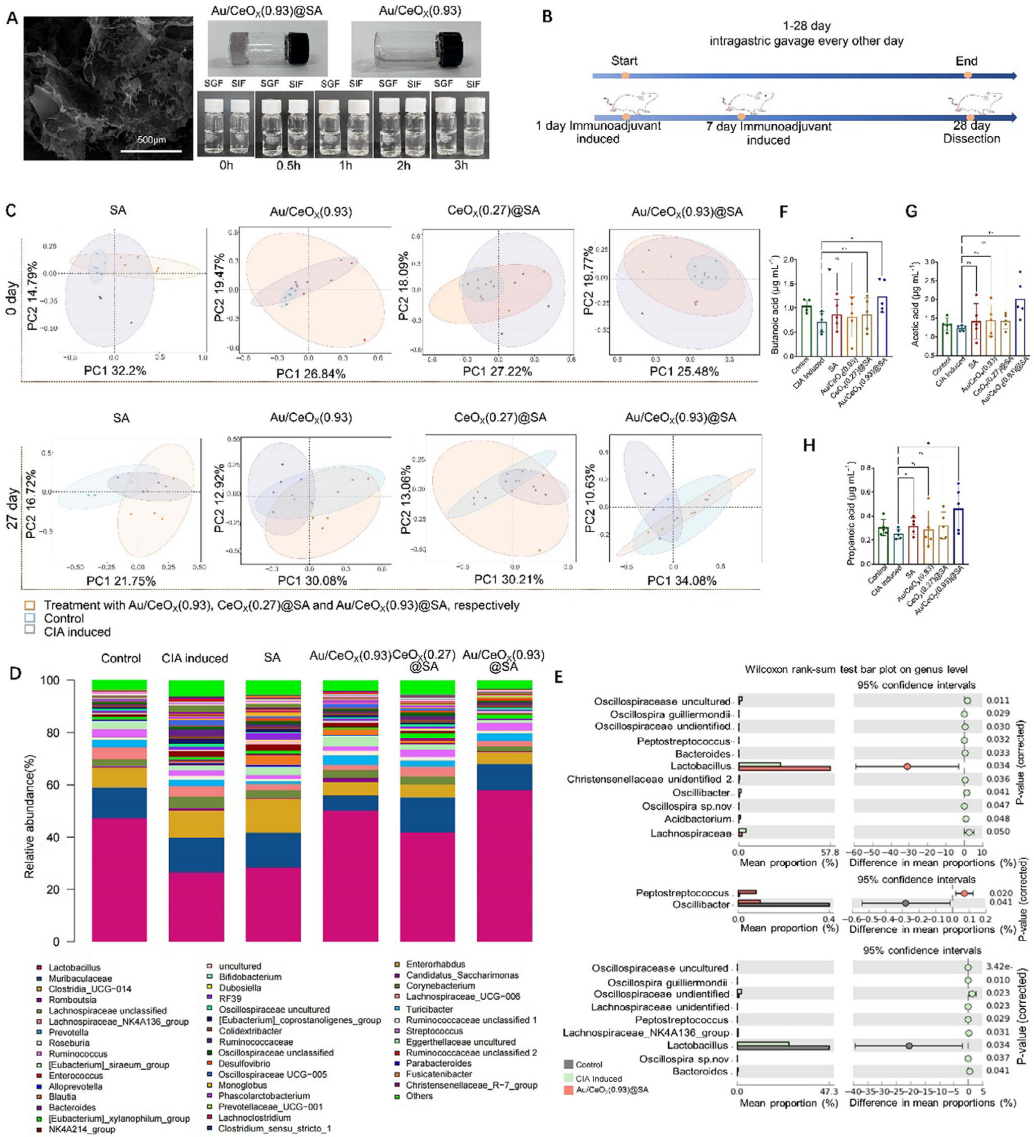
A) Left: Scanning electron microscopy imaging of calcium ions crosslinked SA when crosslinking with Au/CeO_X_(0.93); Upper right: gel‐like Au/CeO_X_(0.93)@SA and aqueous Au/CeO_X_(0.93); Low right: solubility evaluation of Au/CeO_X_(0.93)@SA when immersing them in simulated gastric fluid (SGF, pH≤2.0) and simulated intestinal fluid (SIF, pH ≥ 5.5) for 0–3 h; B) The entire process involved the induction of double immunization in rats and the oral administration of Au/CeO_X_(0.93)@SA over a 28‐day period; C) The β diversity analysis of the gut microbiota in 1st and 27th day period by administration of SA, Au/CeO_X_(0.93), Au/CeO_X_(0.27)@SA, and Au/CeO_X_(0.93)@SA, respectively; D) The composition analysis of gut microbiota at the genus level through bar plot indicating the relative abundance of dominant genera (>0.02%) by oral administration of SA, Au/CeO_X_(0.93), Au/CeO_X_(0.27)@SA, and Au/CeO_X_(0.93)@SA, respectively; E) The analysis of the differential species of gut microbiota at the genus level among control groups, CIA‐induced groups and Au/CeO_X_(0.93)@SA groups; F) Metabolomics analysis of short‐chain fatty acids in feces/mg (acetic acid, butanoic acid and propanoic acid) by oral administration of SA, Au/CeO_X_(0.93), Au/CeO_X_(0.27)@SA, and Au/CeO_X_(0.93)@SA, respectively. Data are presented as means ± SD (*n* = 3). One‐way ANOVA with Tukey's multiple comparisons test, *p < 0.05, **p < 0.01.

For effective regulation of the intestinal environment and the symbiotic microbiota, it is imperative that the nanomedicine administered orally remain intact as they pass through the stomach and then undergo sustained‐release transformation within the intestinal tract. Therefore, we cross‐linked the acid‐resistant SA with the Au/CeO_X_(0.93) to prepare Au/CeO_X_(0.93)@SA (**Figure** **4**A left). As shown in Figure 4A (right), no obvious change was observed in gel‐like Au/CeO_X_(0.93)@SA after 3 h treatment in artificial simulated gastric fluid (SGF, pH ≈1.5). In contrast, the Au/CeO_X_(0.93)@SA could gradually swell and almost completely degrade after 3 h suspension in artificial intestinal fluid (pH 5.5–7.4). Therefore, the Au/CeO_X_(0.93)@SA were anticipated to exert therapeutic effects by being absorbed into the intestinal tract after oral administration. To further investigate the effects of orally administered Au/CeO_X_(0.93)@SA on the microbiota‐gut‐joint axis, we utilized a classic rat model of RA with collagen induced arthritis (CIA) and Freund's adjuvant. For a rigorous comparative treatment evaluation, two additional administration groups were employed: Au/CeO_X_(0.93) without SA protection, and CeO_X_(0.27) encapsulated by SA hydrogel. Considering prior studies have documented that SA alone can modulate the gut microbiota,^[^ ^30^, ^31^ ^]^ we compared CeO_X_(0.27) and Au/CeO_X_(0.93) under identical SA encapsulation for conducting a rigorous in vivo assessment of their impact on the microbiota‐gut‐joint axis. The entire process involved the oral administration of Au/CeO_X_(0.93)@SA over a 27day period (Figure 4B), because compared with the CIA‐induced group, RA will be significantly relieved after 27 days. The oral administration process did not induce any damage to the heart, liver, spleen, lungs, kidneys, or other organs in RA rats (Figure S11, Supporting Information), demonstrating the excellent tissue biocompatibility of all the Au/CeO_X_(0.93), CeO_X_(0.27)@SA, and Au/CeO_X_(0.93)@SA nanozymes.

The diversity of gut microbiota in rats was assessed through 16S rRNA sequencing of fecal samples. At the genus level, α‐diversity metrics, including Shannon index, Faith's PD, and Pielou's evenness, remained statistically unchanged (Figure S12, Supporting Information). In contrast, β‐diversity analyses revealed a pronounced shift in community structure, as evidenced by principal coordinates analysis (PCoA) and analysis of similarities (ANOSIM). The analysis compared samples collected on the 0 and 27th days from rats administered with Au/CeO_X_(0.93), CeO_X_(0.27)@SA, and Au/CeO_X_(0.93)@SA, respectively. The results are presented in Figure 4C. During the initial phase, the sample points for each group exhibited a relatively clustered distribution. This clustering indicates a high degree of similarity in the community structure of the gut microbiota among rats before immune induction. This suggests that at this stage, the community structure of the gut microbiota was relatively consistent across all groups. On the 27th day, a decrease in sample similarity was observed between the CIA‐induced group and the control group. At the same time, the sample points of the Au/CeO_X_(0.93)@SA group were relatively aggregated and positioned at a distance from the CIA induced group, similar to a distinct profile of the control group. In contrast, the Au/CeO_X_(0.93) group and CeO_X_(0.27)@SA group were still unable to successfully restore the gut microbiota similarity to that of the health control group. This indicates that the Au/CeO_X_(0.93)@SA group exhibited a distinct profile that positively regulated the imbalance of the intestinal flora, facilitating the restoration of the microbiota structure toward a normal state.

Figure 4D presents a detailed stacked column chart analyzing the relative abundance of various microbial communities across different samples, supported by the results from the Statistical Analysis of Taxonomic and Functional Profiles (STAMP) shown in Figure 4E. The Venn diagram in Figure S13 (Supporting Information) analyzed the operational taxonomic units of the gut microbiota at the genus level. These analysis results all underscore the superior efficacy of the Au/CeO_X_(0.93)@SA treatment in restoring intestinal flora balance. When comparing the control group to the CIA‐induced group, we identified significant differences in nine bacterial genera, underscoring the profound influence of RA development on gut microbiota. Further analysis revealed that the Au/CeO_X_(0.93)@SA group exhibited significant differences in the relative abundance of 11 distinct bacterial genera when compared to the CIA‐induced group. This suggests that Au/CeO_X_(0.93)@SA treatment significantly influences microbial community structure. Importantly, no significant differences were found between the control group and the Au/CeO_X_(0.93)@SA group in terms of bacterial genera. These findings collectively suggest that Au/CeO_X_(0.93)@SA may play a crucial role in modulating gut microbiota, particularly under conditions that disrupt microbial balance, such as those induced by CIA. This treatment appears to restore or maintain a microbial community structure akin to that of the healthy control group. The decline in intestinal obligate anaerobes indirectly indicates intestinal oxygen leakage, which could disrupt the intestinal oxidative stress microenvironment.^[^ ^32^ ^]^ Upon effectively reducing excessive ROS by Au/CeO_X_(0.93)@SA and minimizing oxygen generation in the intestinal tract, the microenvironment becomes anaerobic, allowing obligate anaerobes to recover to levels similar to the control group.

Our findings indicate that *Lactobacillus* is the most differentially abundant genus, comprising 40%–60% of the microbial community in each sample. This genus is strongly associated with the short‐chain fatty acid (SCFA) metabolism of gut microbiota, primarily due to its ability to ferment undigested carbohydrates, thereby producing SCFAs.^[^ ^33^ ^]^ As the dominant microbial metabolites, SCFAs integrate the gut‐joint axis in RA by simultaneously fortifying epithelial barrier integrity, rebalancing immunity, and restraining osteoclastic bone resorption.^[^ ^34^, ^35^ ^]^ In Figure 4F–H, SCFA levels in the RA model group are markedly lower than those in the normal group, which were consistent with previous reports.^[^ ^36^ ^]^ Following the active intervention of Au/CeO_X_(0.93)@SA on intestinal dysbiosis, the levels of signature microbial SCFAs‐acetate, propionate, and butyrate‐rose markedly, sharply contrasting with their significant reduction in CIA model rats and, moreover, surpassing those in the normal control group (Figures 4F–H). Although SA alone can elevate SCFA levels,^[^ ^30^, ^31^ ^]^ a head‐to‐head comparison of Au/CeO_X_(0.93)@SA and CeO_X_(0.27)@SA, each loaded with an identical SA dose, demonstrates that the former elicits a markedly stronger modulation of SCFA metabolism. This superiority arises from the intrinsically higher catalytic reactivity of Au/CeO_X_(0.93) rather than from a differential SA contribution. These superior SCFA rebound underpins the concomitant attenuation of intestinal inflammation, restoration of epithelial tight‐junction architecture, and downstream dampening of autoimmune joint destruction.

### 2.6 Anti‐Rheumatoid Arthritis Efficacy Evaluation of the Au/CeO_X_(0.93)@SA

**Figure 5:**
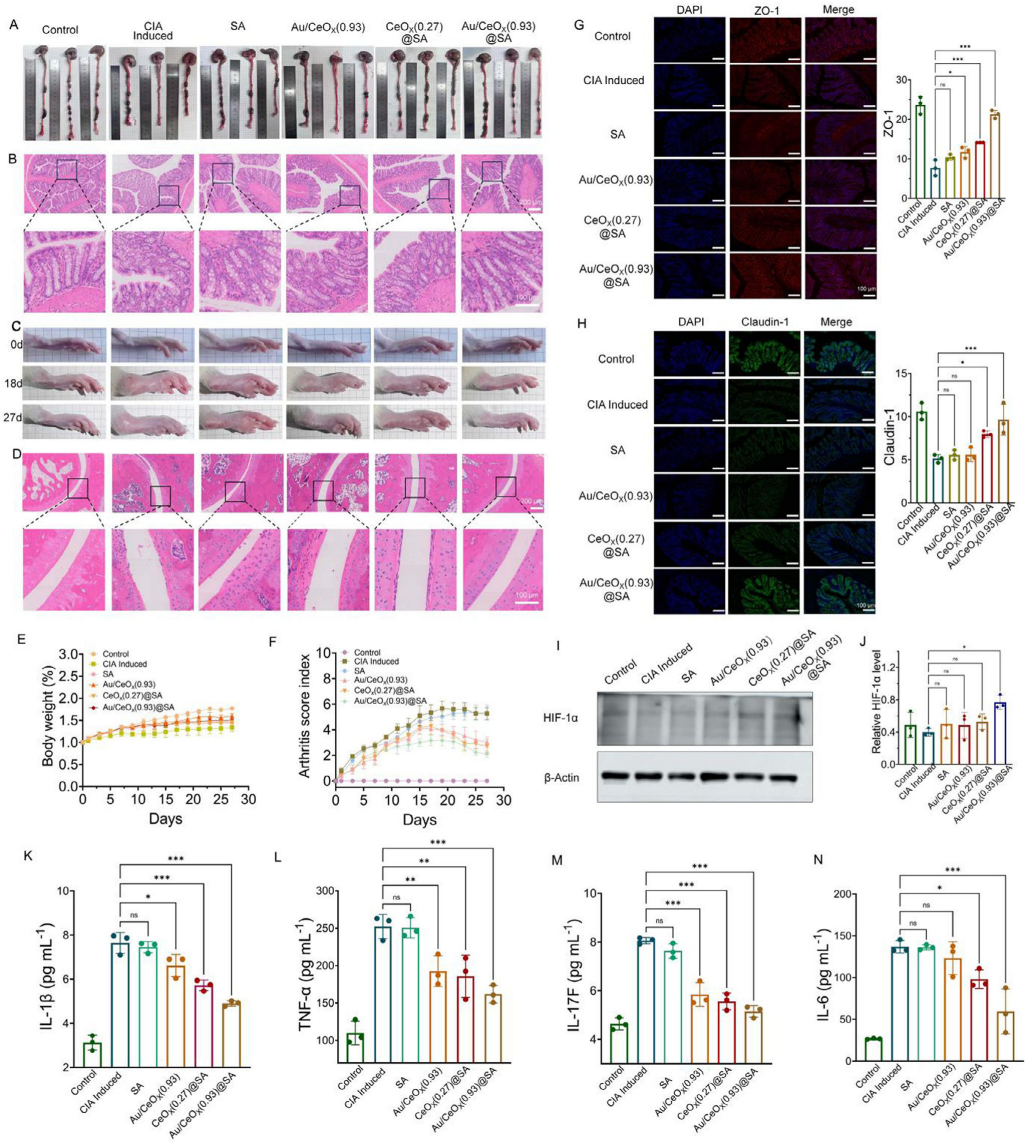
Pathological changes in the colon and joints tissues of RA rats by oral administration of SA, Au/CeO_X_(0.93), Au/CeO_X_(0.27)@SA, and Au/CeO_X_(0.93)@SA, respectively. A) The comparison of colon length; B) H&E stained images of colonic tissues; C) Representative images of hind paws from rats in each group throughout the entire treatment period; D) H&E stained images of ankle joint tissues; E) Comparison of body weight in each group of rats; F) Comparison of ankle arthritis swelling scores in each group of rats. Data are presented as means ± SD (*n* = 5); G) Immunofluorescence staining (left) and fluorescence intensity analysis(right) of colonic tissues indicated ZO‐1 (red signal). Blue signal represents nuclear staining with DAPI. Data are presented as means ± SD (*n* = 3); H) Immunofluorescence staining (left) and fluorescence intensity analysis(right) of colonic tissues indicated Claudin‐1 (green signal). Blue signal represents nuclear staining with DAPI. Data are presented as means ± SD (*n* = 3); I) The expression levels of HIF‐1α in colonic tissues by Western Blot analysis and J) their gray‐scale analysis. Data are presented as means ± SD (*n* = 3); (K‐N) Concentrations of pro‐inflammatory cytokines (IL‐1β, IL‐6, IL‐17F, TNF‐α) in serum. Data are presented as means ± SD (*n* = 3). One‐way ANOVA with Tukey's multiple comparisons test, *p < 0.05, **p < 0.01, and ***p < 0.001.

The in vivo anti‐inflammatory efficacy of Au/CeO_X_(0.93)@SA was further evaluated in **Figure** **5**. Given that RA may originate from gut microbiota dysbiosis, causing intestinal inflammation and subsequent systemic inflammatory responses that drive joint destruction, colon length and its inflammatory pathological conditions were assessed as the initial indicators of the efficacy of Au/CeO_X_(0.93)@SA in alleviating intestinal inflammation. As shown in Figure 5A, the colon length in the CIA‐induced group was significantly reduced compared to the control group. Hematoxylin and eosin (H&E, Figure 5B) staining revealed extensive infiltration of inflammatory cells and irregular, shallower crypt structures in the colonic tissue of the CIA‐induced RA group. These findings indicate that the CIA‐induced model not only induces localized inflammation in the ankle joint but also causes significant intestinal lesions and inflammation. In contrast, oral administration of Au/CeO_X_(0.93)@SA significantly restored colon length to levels comparable to those of the control group. H&E staining further demonstrated that Au/CeO_X_(0.93)@SA treatment improved the irregularity of colonic crypt structures and markedly reduced inflammatory cell infiltration. Correspondingly, the ankle joints of CIA‐induced RA rats exhibited noticeable redness and swelling (Figure 5C,D). On day 18, the degree of foot swelling in the CIA‐induced group peaked, consistent with the arthritis score based on hind paw swelling. Notably, the degree of foot swelling was significantly lower in the Au/CeO_X_(0.93)@SA treatment group compared to the CIA‐induced group, with no obvious swelling observed throughout the experiment, indicating a significant suppression of arthritis onset. Histopathological analysis of the ankle joints revealed severe synovial hyperplasia and substantial inflammatory cell infiltration in the CIA‐induced group, with irregularities observed on the cartilage surface. In contrast, the Au/CeO_X_(0.93)@SA group showed a significant reduction in inflammatory cells and intact cartilage tissue, consistent with the arthritis score in Figure 5F and Table S2 (Supporting Information).

To deeply investigate the regulatory effect of Au/CeO_X_(0.93)@SA on the intestinal barrier function in RA rats, the expression levels of intestinal epithelial tight junction proteins, such as Claudin‐1 and ZO‐1, were analyzed by immunofluorescence (Figure 5G,H). The fluorescence intensities of Claudin‐1 and ZO‐1 in the colonic tissues of the RA model were lower than those in the control group. This may be attributed to the disorder of the intestinal flora in the RA model rats, which increases intestinal permeability and down‐regulates tight junction protein expression. In contrast, the fluorescence intensities of ZO‐1 and Claudin‐1 in the colonic tissues of RA model rats following treatment were higher than those in the CIA‐induced group. Notably, the Au/CeO_X_(0.93)@SA treatment group exhibited significantly elevated levels of tight junction proteins, which played a crucial role in repairing intestinal barrier function. This not only effectively reduced intestinal permeability but also alleviated the systemic inflammatory response induced by intestinal barrier dysfunction. Another key aspect of evaluating intestinal barrier function recovery is assessing the restoration of the intestinal hypoxic environment. As the aforementioned findings indicate, the recovery of anaerobes following Au/CeO_X_(0.93)@SA treatment could be associated with this modulation. To investigate this, we measured the expression levels of hypoxia‐inducible factor‐1α (HIF‐1α), a key marker molecule for measuring the oxygen level in intestinal tissues.^[^ ^37^ ^]^ As shown in Figures 5I,J and S14 (Supporting Information), HIF‐1α expression was significantly lower in RA rats compared to the control group. Oral administration of Au/CeO_X_(0.93) and CeO_X_(0.27)@SA increased HIF‐1α levels but did not restore them to normal. In contrast, oral administration of Au/CeO_X_(0.93)@SA significantly elevated HIF‐1α expression, surpassing that of the control group. This suggests that the restored hypoxic microenvironment supports the normal growth of intestinal symbiotic flora, consistent with our gut microbiota analysis.

The efficacy of Au/CeO_X_(0.93)@SA in modulating inflammation was further demonstrated by detecting changes in the levels of pro‐inflammatory cytokines in the serum using enzyme‐linked immunosorbent assay (ELISA). As shown in Figure 5K–N, compared with the blank group, serum levels of TNF‐α, IL‐6, IL‐17F, and IL‐1β were significantly upregulated in the CIA‐induced group, indicating a pronounced inflammatory response. Although the levels of inflammatory factors in the Au/CeO_X_(0.93) and CeO_X_(0.27)@SA treatment groups decreased to some extent, the effect was not significant. In contrast, the Au/CeO_X_(0.93)@SA group exhibited extremely significant differences in the levels of pro‐inflammatory factors such as TNF‐α, IL‐17F, IL‐6, and IL‐1β compared with the induced group (*p *< 0.001). These results demonstrate that Au/CeO_X_(0.93)@SA effectively inhibits the inflammatory response in RA and reduces the levels of pro‐inflammatory factors, highlighting its potential as an effective material for inhibiting the onset of RA.

## 3 Conclusion

In summary, a classical valence state regulation strategy based on the Au deposition method was employed to systematically investigate the correlation between doping concentration and *V_O_* concentration. This approach aims to maximize the proportion of Ce^3+^ to achieve highly efficient OXD‐, SOD‐, and POD‐like activities while suppressing CAT‐like activity associated with Ce^4+^. HAADF‐STEM and X‐ray absorption spectroscopy confirm the successful incorporation of Au into the lattice of CeO_X_. Catalytic activity studies and DFT calculations demonstrate that Au doping lowers the activation energy barriers of O_2_^•−^→H_2_O_2_→OH^−^ cascade reaction and enhances catalytic efficiency via Au‐Ce interface interactions, thereby boosting the OXD‐SOD‐POD self‐cascade enzyme‐like activity of CeO_X_ nanozymes. Then, optimal Au/CeO_X_(0.93) was encapsulated within SA to construct a gastric acid‐resistant system (Au/CeO_X_(0.93)@SA) that possesses active targeted decomposition ability in the intestinal pathological microenvironment of RA. Upon oral administration to a rat model of RA, Au/CeO_X_(0.93)@SA efficiently modulated the gut microbiota for early RA intervention. The disordered gut microbiota were restored to near‐normal levels, with normalized short‐chain fatty acid metabolism, which significantly inhibiting RA progression. In summary, Au/CeO_X_(0.93)@SA harnesses single‐enzyme cascade catalysis and microenvironment adaptability to target ROS in RA, establishing a generalizable paradigm for intestinal‐microenvironment‐responsive scavengers that can intercept other gut–organ axis inflammatory pathologies at an early stage.

## 4 Experimental Section

### Materials

Cerium(III) acetate hydrate, oleylamine, xylene, chloroauric acid, ethylene glycol bis (2‐aminodiethyl ether) tetraacetic acid (EGTA), diethylenetriaminepentaacetic acid (DTPA), dimethyl‐1‐pyrroline N‐oxide (DMPO), hypoxanthine, xanthine oxidase, bovine type‐II collagen, SA, TMB, OPD, and LPS were obtained from Shanghai Macklin Biochemical Co., Ltd. (Shanghai, China). DCFH‐DA, DHE, HPF, and [Ru(dpp)_3_]^2+^ fluorescent probe were obtained from MedChemexpress Co., Ltd. (Shanghai, China). ROSGreen H_2_O_2_ probe was obtained from Shanghai Maokang Biotechnology Co., Ltd. (Shanghai, China). Complete Freund's adjuvant (CFA) and incomplete Freund's adjuvant (IFA) were obtained from Beyotime Biotech. Inc. (Shanghai, China).

### Cell line and Animals

The cell lines in this study were all obtained from Cell Bank, Shanghai Institute of Biochemistry and Cell Biology, and cultured in high glucose Dulbecco's modified eagle's medium (Gibco, China) supplemented with 10% fetal bovine serum (Adamas life, South America) and 1% penicillin/streptomycin at 37 °C with 5% CO_2_ atmosphere. Male Wistar rats (6–8 weeks old) were purchased from SPF (Beijing) Biotechnology Co., Ltd (Beijing, China) and kept under specific pathogen‐free conditions throughout the experiment. All animal experiments followed guidelines approved by the ethics committee of Xinxiang Medical University.

### Preparation of Au/CeO_X_ with Different Ce^3+^/Ce^4+^ Ratio

1) 1 mM cerium(III) acetate hydrate and 12 mm oleylamine were added to 15 mL xylene, stirred at room temperature for 12 h, and heated under vacuum to 90 °C (2 °C min^−1^). At 90 °C, 1 mL deionized water was added, causing the solution to turn light grey. The mixture was then heated at 90 °C for 3 h to form a pale yellow colloidal solution. After cooling to room temperature, the cerium oxide were precipitated, washed with ethanol and acetone, centrifuged at 10 000 r min^−1^ for 10 min, and dried in a vacuum oven at 45 °C for 12 h.^[^ ^38^, ^39^ ^]^ 2) 20 mg of pristine CeO_X_ was weighed, dispersed in 20 mL deionized water, and ultrasonicated for 10 min to ensure homogeneity. The pH was adjusted to 10 using 1 m sodium hydroxide, and different volumes of chloroauric acid solution were added to prepare Au/CeO_X_ nanozymes with varying doping levels. The mixture was stirred at room temperature for 4 h, 10 000 r min^−1^ centrifuged for 10 min, and dried in a vacuum oven at 60 °C for 3 h.

### Preparation of CeO_X_(0.27)@SA and Au/CeO_X_(0.93)@SA

300 mg of SA and 10 mg of CeO_X_(0.27) or Au/CeO_X_(0.93) were dissolved in 10 mL deionized water and thoroughly mixed. A Ca^2+^‐EGTA solution, prepared by dissolving equimolar amounts of CaCl_2_ and EGTA in water and adjusting the pH to 7 with 1 m NaOH, was then added. The Ca^2+^‐EGTA solution was formulated with a Ca^2+^:GDL molar ratio of 1:2. Upon the addition of GDL, Ca^2+^ ions were slowly released from the Ca^2+^‐EGTA complex, triggering the gelation of the alginate solution. The mixture was allowed to stand for at least 24 h to ensure complete gelation.

### Characterization of Au/CeO_X_@SA and Au/CeO_X_

XPS measurements were performed with Thermo Scientific Escalab 220i‐XL. HAADF‐STEM measurements were performed on a JEOL JEMARM200F STEM/TEM. XRD spectra were collected by PANalytical X'Pert Pro Diffractometer with Cu Kα radiation (45 kV, 40 mA). XAS were recorded on BL11B beam line at the Shanghai Synchrotron Radiation Facility (SSRF). The cyclic voltammetry of redox potential was evaluated by a bipotentiostat (Corrtest, CS2350M).

### Detection of SOD‐like Enzymes

The ability of Au/CeO_X_(0.93) to eliminate •OH or O_2_^−•^ was measured by ESR (Bruker, EMXplus). The O_2_^•−^ generation system was established as follows: A centrifuge tube was sequentially filled with 70 µL of 25 mm diethylenetriaminepentaacetic acid (DTPA) in phosphate buffer, 20 µL of 1 m 5,5‐Dimethyl‐1‐pyrroline N‐oxide (DMPO), and 100 µL of 1 mm hypoxanthine. The reaction was initiated by adding 10 µL of xanthine oxidase (1 U mL^−1^), followed by vortexing. The different samples were then added, vortexed for 30 s, and transferred to an ESR tube for analysis.

For the •OH detection system: 70 µL of 0.735 mM FeSO_4_, 100 µL of the sample, and 25 µL of 0.315 mm H_2_O_2_ were mixed, shaken for 1 min, and 2.26 µL of 1 m DMPO was added. The solution was then transferred to an ESR tube for detection.

### Detection of CAT‐like Enzymes

The dissolved oxygen analyzer (Vernier, DO‐BTA Dis) was employed to evaluate the oxygen‐generation capabilities of various samples reacting with H_2_O_2_. PBS solutions at pH 5.5 and pH 7.4 were prepared and stabilized for 300 s and mixed with 100 mm H_2_O_2_. Samples were added to each mixture to ensure a total volume of 200 mL. Dissolved oxygen levels were continuously measured for 1 000 seconds after stabilization to assess the oxygen‐evolving performance of the samples.

### Detection of POD‐like Enzymes

The POD‐like activity of the samples was assessed using TMB as the substrate. In a typical assay, 100 µL of 10 mm TMB solution, 100 µL of 100 mm H_2_O_2_ solution, 650 µL of deionized water, and 50 µL of a 100 µg mL^−1^ sample solution were mixed together in the presence of 100 µL of PBS. The mixture was allowed to react for 30 min, after which the absorbance at 652 nm was measured using a UV–Vis spectrophotometer (Beijing Purkinje, TU‐1900) to quantify the extent of TMB oxidation, indicative of the POD‐like activity.

### Detection of OXD‐like Enzymes

The OXD‐like activity of the samples was evaluated using o‐phenylenediamine dihydrochloride (OPD) as the substrate. In a typical assay, 3 mg of OPD was dissolved in 980 µL of the sample solution with a concentration of 400 µg mL^−1^. The mixture was incubated for 30 min at room temperature to allow the enzymatic reaction to proceed. Subsequently, the absorbance at 426 nm was measured using a UV–Vis spectrophotometer (Beijing Purkinje, TU‐1900) to quantify the OXD‐like activity of the samples.

### In Vitro Cytotoxicity Assay and Intracellular ROS Detection

The cytotoxicity of Au/CeO_2_(0.93)@SA on HepG‐2, HaCaT and BRL3A was detected by MTT assay. Intracellular levels of ROS, O_2_^•−^ and •OH were measured using the DCFH‐DA, DHE and HPF fluorescent probe. RAW264.7 cells were seeded into a confocal dish at a density of 1 × 10⁴ cells per well and cultured for 24 h. Subsequently, the cells were stimulated with LPS for an additional 12 h. Meanwhile, the cells were also treated by CeO_X_(0.27)@SA, Au/CeO_X_(0.93) and Au/CeO_X_(0.93)@SA for 12 h respectively. The cells were then gently washed with PBS, and the fluorescence signal was measured using a fluorescence confocal microscope (ZEISS, LSM900).

### The Ratio of Macrophage Polarization Phenotype was Detected by Flow Cytometry

The M1/M2 macrophage polarization phenotypes were analyzed by flow cytometry (BD, FACSAriaII). Briefly, RAW264.2 cells were induced by LPS and incubated with the samples for 24h. Then, the cells were digested, washed and collected. Single cell suspension was centrifuged (800 g, 5 min) to remove the supernatant. Hundred microliter of Cell suspension and fluorescent‐labeled CD206 (Biolegend, 141733) and CD86 (Biolegend, 105045) antibodies were added subsequently. After washing, 150 µL of PBS was added to each tube, and the mixture was detected by flow cytometry.

### RA Animal Models and In Vivo Anti‐RA Efficacy Evaluation

All animal experiments were divided into five groups: PBS, CIA‐induced, oral Au/CeO_X_(0.93), CeO_X_(0.27)@SA, and Au/CeO_X_(0.93)@SA. The RA rats with the CIA model were established by double immunization.^[^ ^40^ ^]^ For the first immunization, rats were injected intradermally at the tail base and right hind foot with an equal‐volume emulsion of bovine type‐II collagen solution and CFA. 7 days later, all rats except the PBS group received a booster immunization with bovine type‐II collagen emulsified in IFA. The oral administration groups (Au/CeO_X_(0.93), CeO_X_(0.27)@SA, and Au/CeO_X_(0.93)@SA) were dosed at 1 mg/kg/day for 27 days via gavage. Body weight and hind paw swelling were measured every 2 days for 28 days and scored on a standard scale. For efficacy evaluation, H&E staining was used to examine ankle joint and colon tissue morphology. Immunofluorescence staining detected ZO‐1 (Abcam, ab307799) and Claudin‐1 (Abcam, ab211737) in colon tissues, while western blot analyzed HIF‐1α (Abcam, ab317044) expression. Serum inflammatory markers (IL‐1β, IL‐6, IL‐17F, and TNF‐α) were assessed using ELISA kits (Ruixin Biotechnology Co., Ltd., Quanzhou, China) according to the manufacturers' instructions.

### 16S rRNA gene Amplicon Sequencing

Genomic DNA was extracted from fecal samples using the Biomiga Stool gDNA Miniprep kit (Biomiga, USA) according to the manufacturer's protocols. The quality and concentration of DNA were determined by 1.0% agarose gel electrophoresis and a NanoDrop ND‐2000 spectrophotometer (Thermo Scientific Inc., USA) and kept at−80 °C prior to further use. The V3–V4 region of the 16S rRNA gene was amplified using primers 338F (5′‐barcode‐ACTCCTACGGGAGGCAGCA‐3′) and 806R (5′‐GGACTACHVGGGTWTCTAAT‐3′).^[^ ^41^ ^]^ Amplicon products were purified, quantified, and sequenced on the Illumina PE300 platform according to the standard protocols by Majorbio Bio‐Pharm Technology Co. Ltd. (Shanghai, China). All statistical analyses were conducted using R software (v4.3.0). Group comparisons were performed using one‐way ANOVA with LSD post‐hoc tests, with statistical significance set at *p*< 0.05. Multivariate analyses including Principal Coordinates Analysis (PCoA), Analysis of Similarities (ANOSIM), Canonical Correspondence Analysis (CCA), and PERMANOVA were implemented through the vegan package. Significant genera in amplicon sequencing were identified using STAMP.^[^ ^42^ ^]^ Data visualization of Venn was generated using R. Heatmap data were log_10_‐transformed prior to visualization.

### Targeted Metabolomics

The GC‐MS metabolomics of the samples was performed using the 8890B‐5977B gas chromatography‐mass spectrometry of Agilent Company analysis by Majorbio Bio‐Pharm Technology Co., Ltd. (Shanghai, China).

### Density Functional Theory (DFT)

The projected augmented wave (PAW) potentials were employed to describe the ionic cores, and a plane‐wave basis set with a kinetic energy cutoff of 450 eV was used to account for the valence electrons.^[^ ^43^, ^44^ ^]^ Partial occupancies of the Kohn−Sham orbitals were allowed using the Gaussian smearing method and a width of 0.02 eV. The electronic energy was considered self‐consistent when the energy change was smaller than 10^−5^ eV. A 1 × 1 × 1 Monkhorst–Pack scheme was used to generate the k‐point grid for the modeled surfaces. A geometry optimization was considered convergent when the force change was smaller than 0.03 eV Å^−1^. Grimme's DFT‐D3 methodology was used to describe the dispersion interactions.^[^ ^45^ ^]^ The gibbs free energy (ΔG) was calculated as follows: ΔG = ΔE+ΔZPE‐TΔS. where E is DFT energy, ZPE is zero point energy, T is temperature (298 K), S is entropy.

## Conflict of Interest

The authors declare have no competing interests.

## Supporting information



Supporting Information

## Data Availability

The data that support the findings of this study are available from the corresponding author upon reasonable request.
