# Supplementary material for: Structure‐Tailoring Cerium Nanozymes with Self‐Cascade ROS Scavenging Catalysis Modulate the Microbiota‐Gut‐Joint Axis for Rheumatoid Arthritis Therapy
Source: Adv Sci (Weinh). 2025 Sep 29;12(47):e12281. doi: 10.1002/advs.202512281 (PMC12713043; doi:10.1002/advs.202512281)
Supplement: Supplementary file 1 — Supporting Information [file ADVS-12-e12281-s001.doc]

Supplemental information

**Structure-Tailoring Cerium Nanozymes with Self-Cascade ROS Scavenging Catalysis Modulate the Microbiota-Gut-Joint Axis for Rheumatoid Arthritis Therapy**

*Ge Wang ^a^, Xueqing Zhang ^a^, Boyuan Zhu ^a^, Suyue Ding ^a^, Dong Yan ^a^, Jing Ma ^c^, Yafang Xiao ^c^, Yafu Wang ^b^, Tianjun Ni ^a^*, Hua Zhang ^b^*, Weisheng Guo ^c^**

Ge Wang, Xueqing Zhang, Boyuan Zhu, Suyue Ding, Dong Yan, Tianjun Ni

^a^ School of Basic Medical Sciences, Xinxiang Medical University; School of Pharmacy, Xinxiang Medical University; The First Affiliated Hospital of Xinxiang Medical University, Xinxiang, 453007, P. R. China.

E-mail: [tjni@xxmu.edu.cn](mailto:tjni@xxmu.edu.cn)

Yafu Wang, Hua Zhang

^b^ Key Laboratory of Green Chemical Media and Reactions, Ministry of Education; Henan Key Laboratory of Organic Functional Molecule and Drug Innovation; School of Chemistry and Chemical Engineering; Henan Normal University, Xinxiang, 453007, P. R. China.

E-mail: [zhh1106@htu.edu.cn](mailto:zhh1106@htu.edu.cn)

Jing Ma, Yafang Xiao, Weisheng Guo

^c^ Department of Minimally Invasive Interventional Radiology, The Second Affiliated Hospital, School of Biomedical Engineering Guangzhou Medical University, Guangzhou 510260, P. R. China.

E-mail: guo_wei_sheng@gzhmu.edu.cn

Keywords: cerium, rheumatoid arthritis, self-cascade, gut-joint axis





Figure S1 XRD patterns of CeO_X_(0.27), Au/CeO_X_(0.49), Au/CeO_X_(0.50), Au/CeO_X_(0.77), and Au/CeO_X_(0.93).


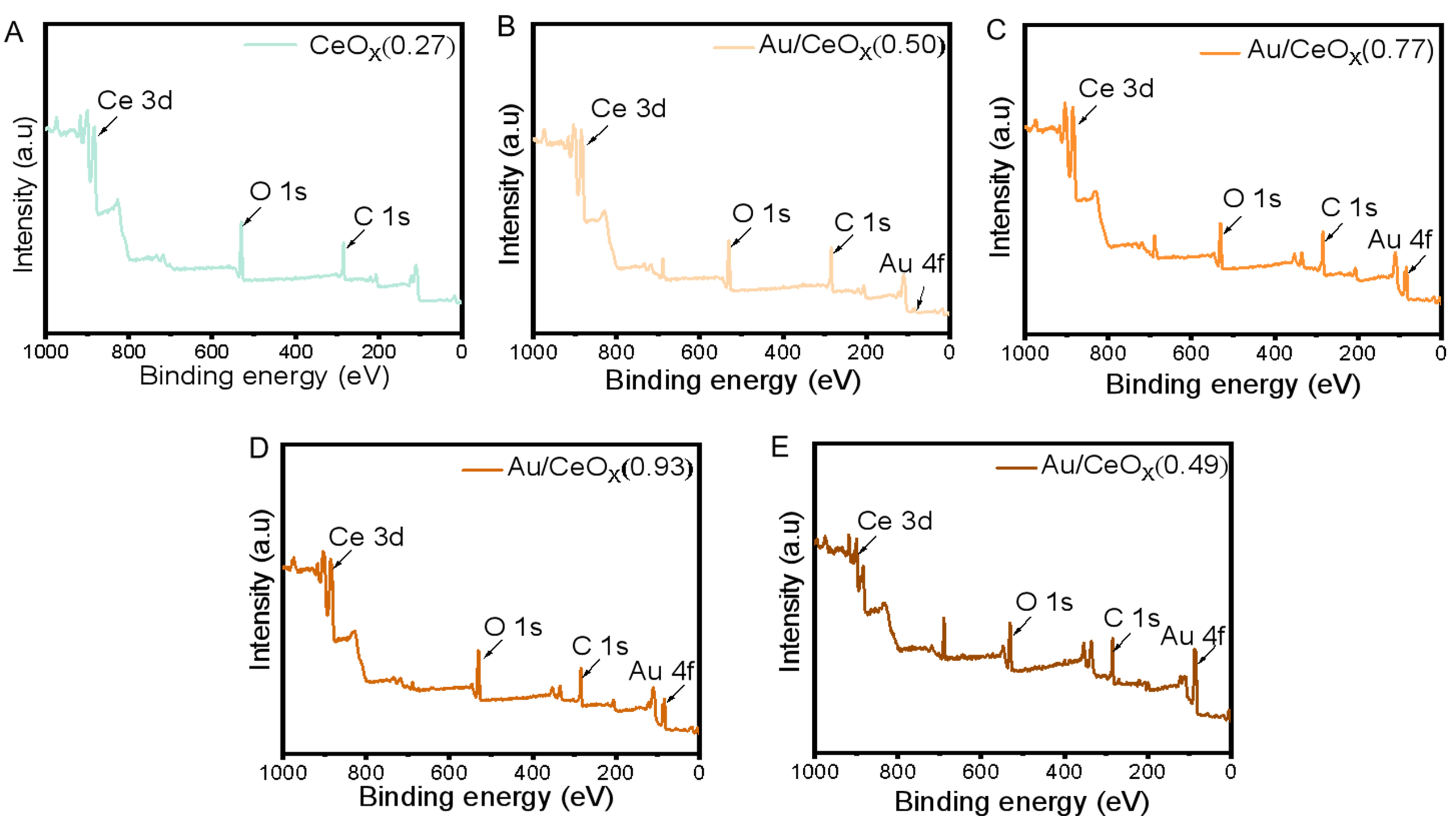


Figure S2 XPS full-spectrum analysis of (A)CeO_X_(0.27), (B)Au/CeO_X_(0.50), (C)Au/CeO_X_(0.77), (D)Au/CeO_X_(0.93) and (E)Au/CeO_X_(0.49).


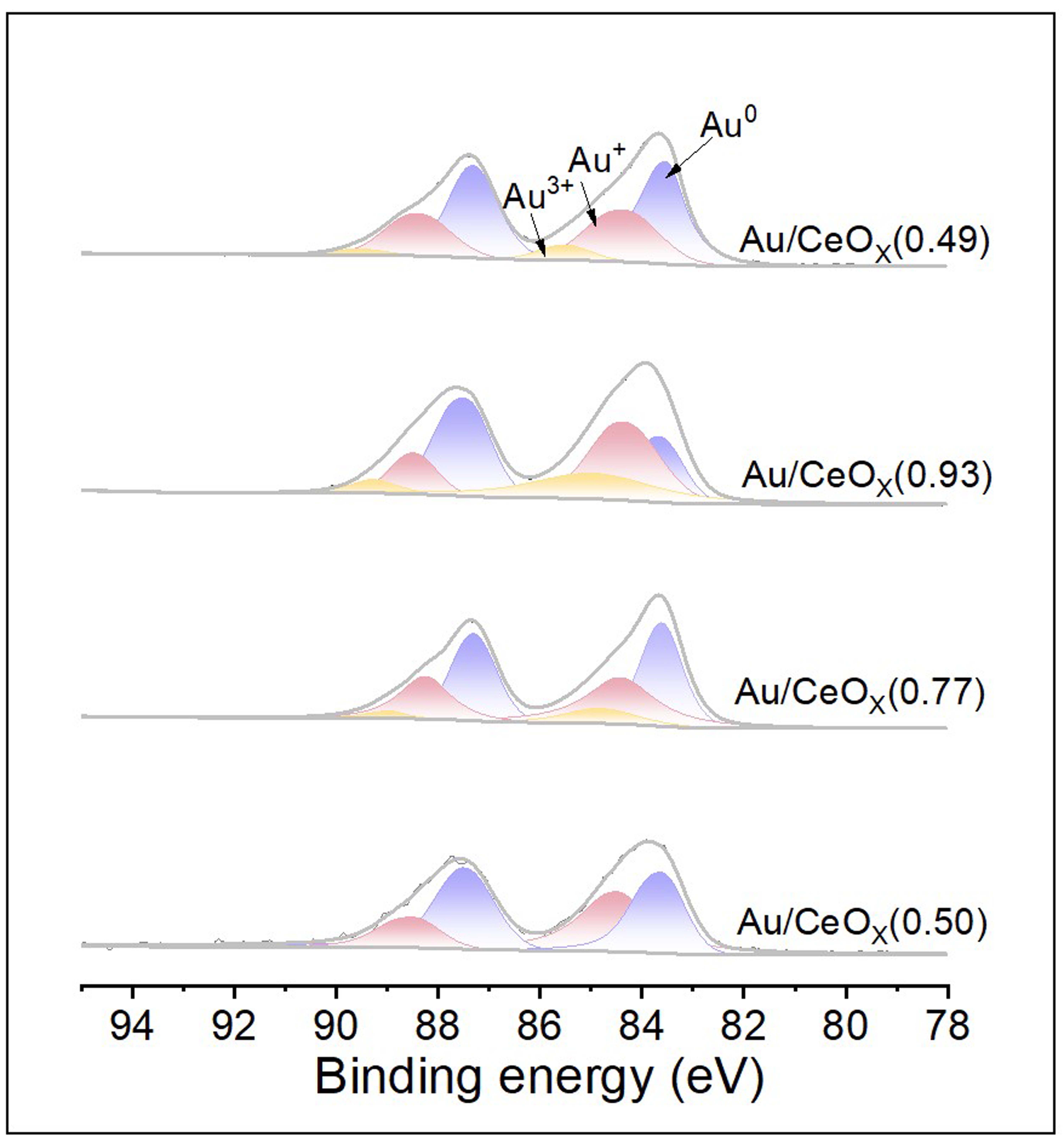


Figure S3 XPS spectra of Au 4f (Au^0^, Au^+^, and Au^3+^)

Table S1 The table for Au^0^, Au^+^, and Au^3+^ comparison of five kinds of CeO_X_ with different valence ratio

|  | Au^0^ | Au^+^ | Au^3+^ |
| --- | --- | --- | --- |
| CeO_X_(0.27) | —— | —— | —— |
| Au/CeO_X_(0.50) | 37.10% | 62.90% | 0% |
| Au/CeO_X_(0.77) | 46.20% | 42.05% | 11.73% |
| Au/CeO_X_(0.93) | 24.88% | 44.66% | 30.45% |
| Au/CeO_X_(0.49) | 55.45% | 36.78% | 7.77% |


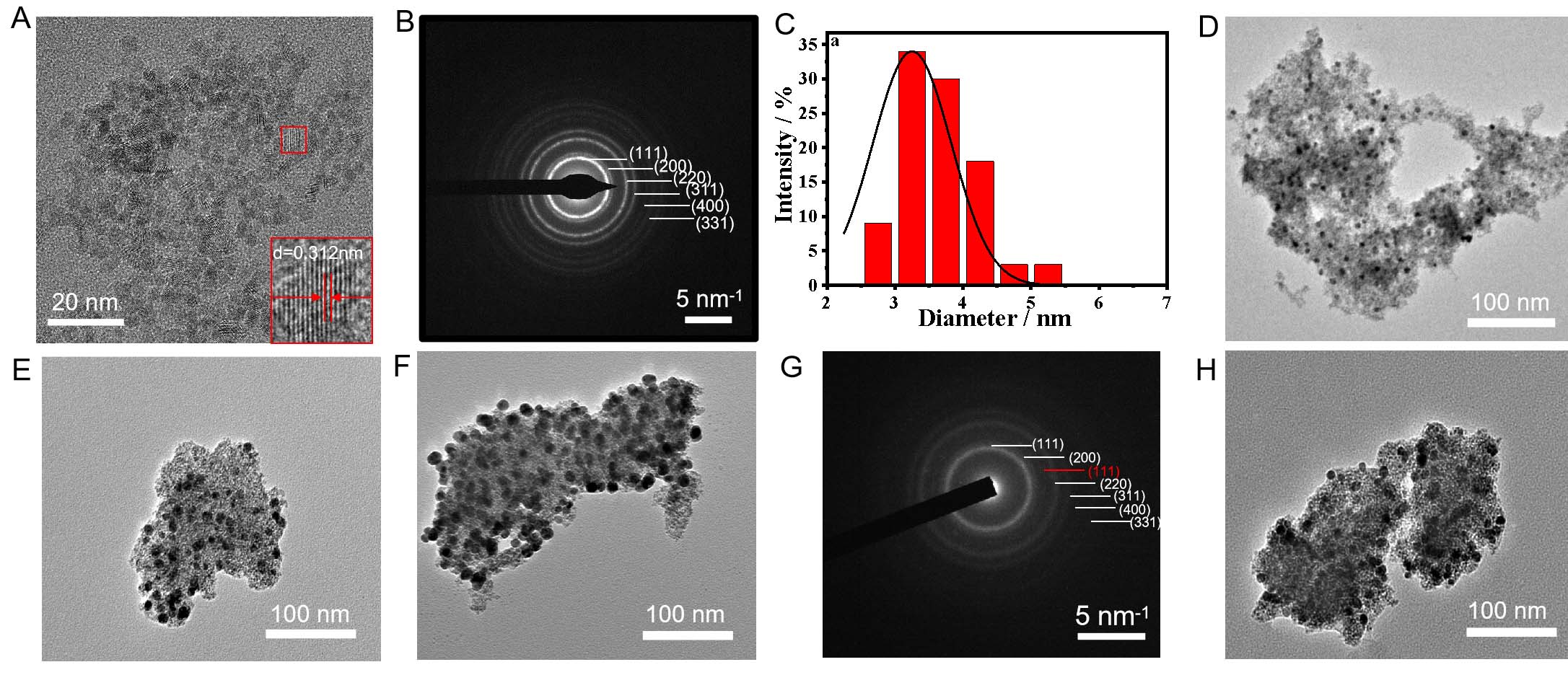
Figure S4 (A)HRTEM images of CeO_X_(0.27); (B)SAED patterns of CeO_X_(0.27); (C)Size distribution analysis of CeO_X_(0.27); (D)HRTEM images of Au/CeO_X_(0.50); (E)HRTEM images of Au/CeO_X_(0.77); (F)HRTEM images of Au/CeO_X_(0.93); (G)SAED patterns of Au/CeO_X_(0.93); (H)HRTEM images of Au/CeO_X_(0.49).


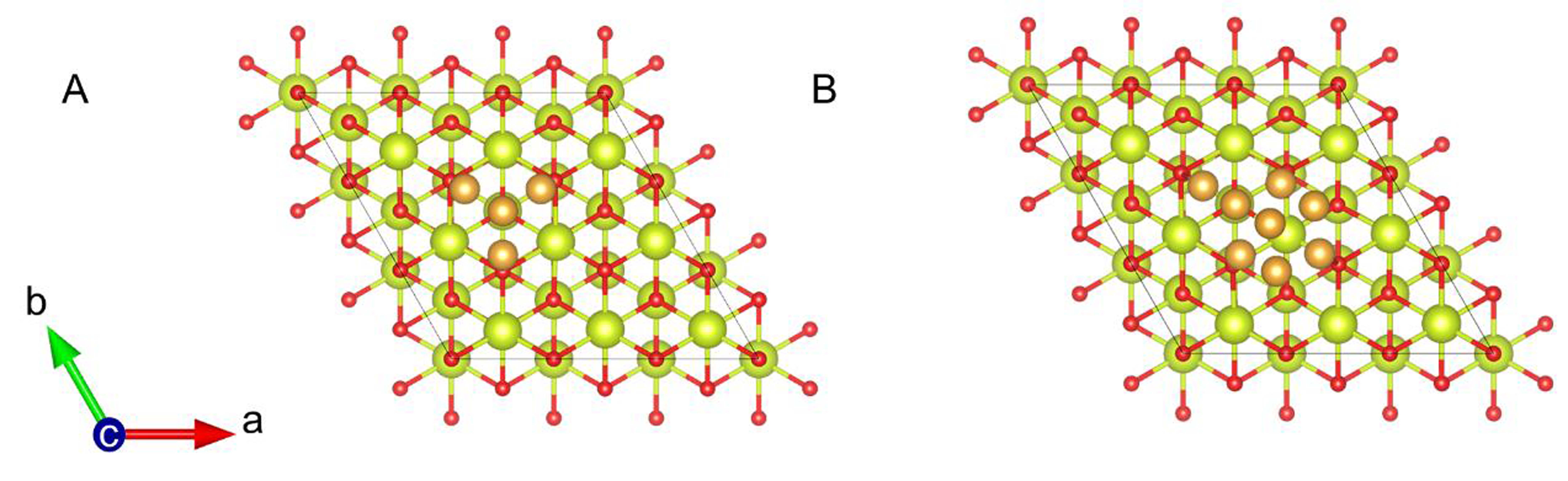
Figure S5 The influence of Au doping on electron transfer in Bader charge analysis. (A) Au/CeO_X_(0.93); (B) Au/CeO_X_(0.49)


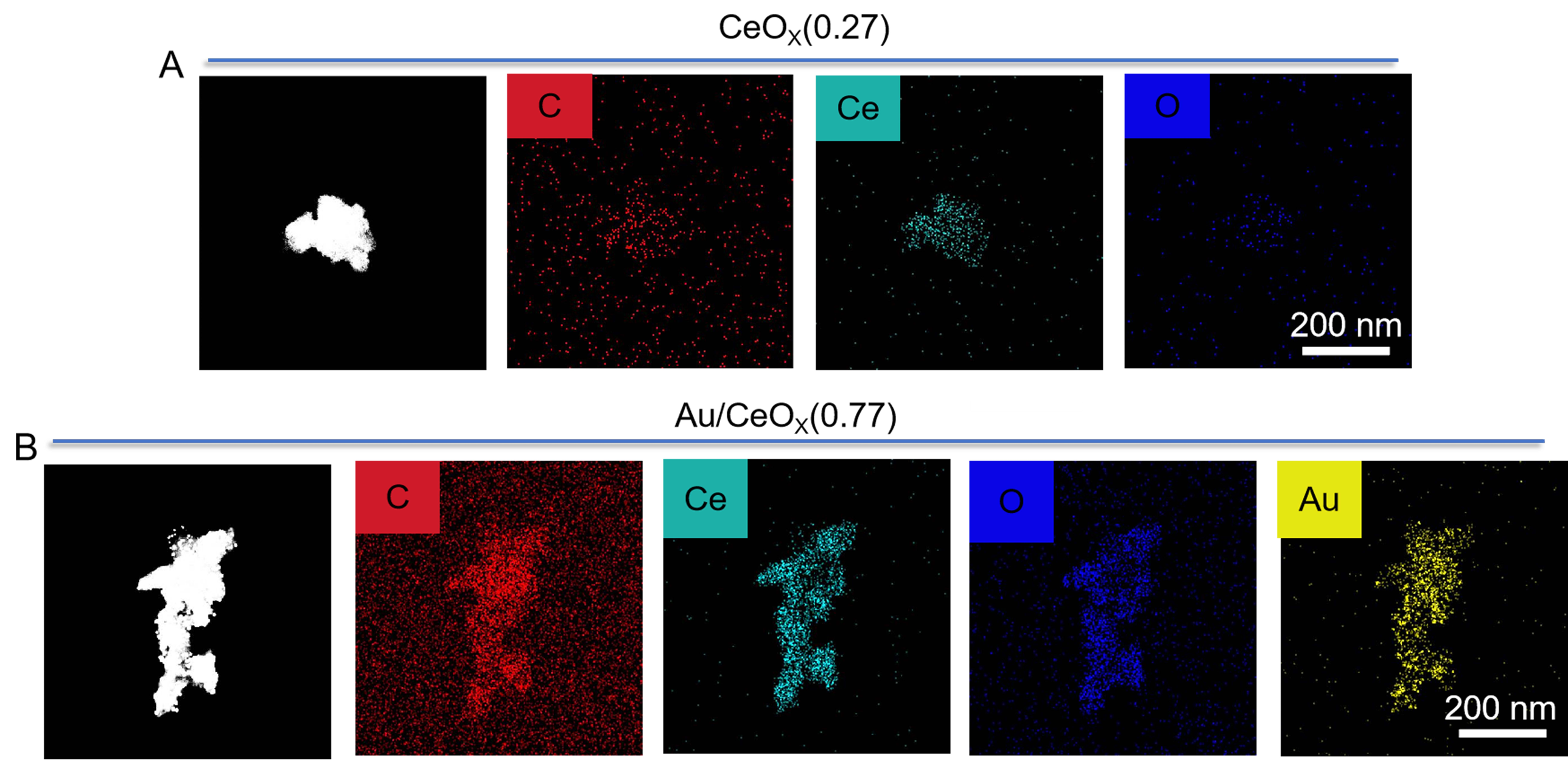


Figure S6 Energy-dispersive spectroscopy mappings of (A)CeO_X_(0.27) and (B)Au/CeO_X_(0.77).





Figure S7 Fourier Transform Infrared Spectroscopy of CeO_X_(0.27) and Au/CeO_X_(0.93).


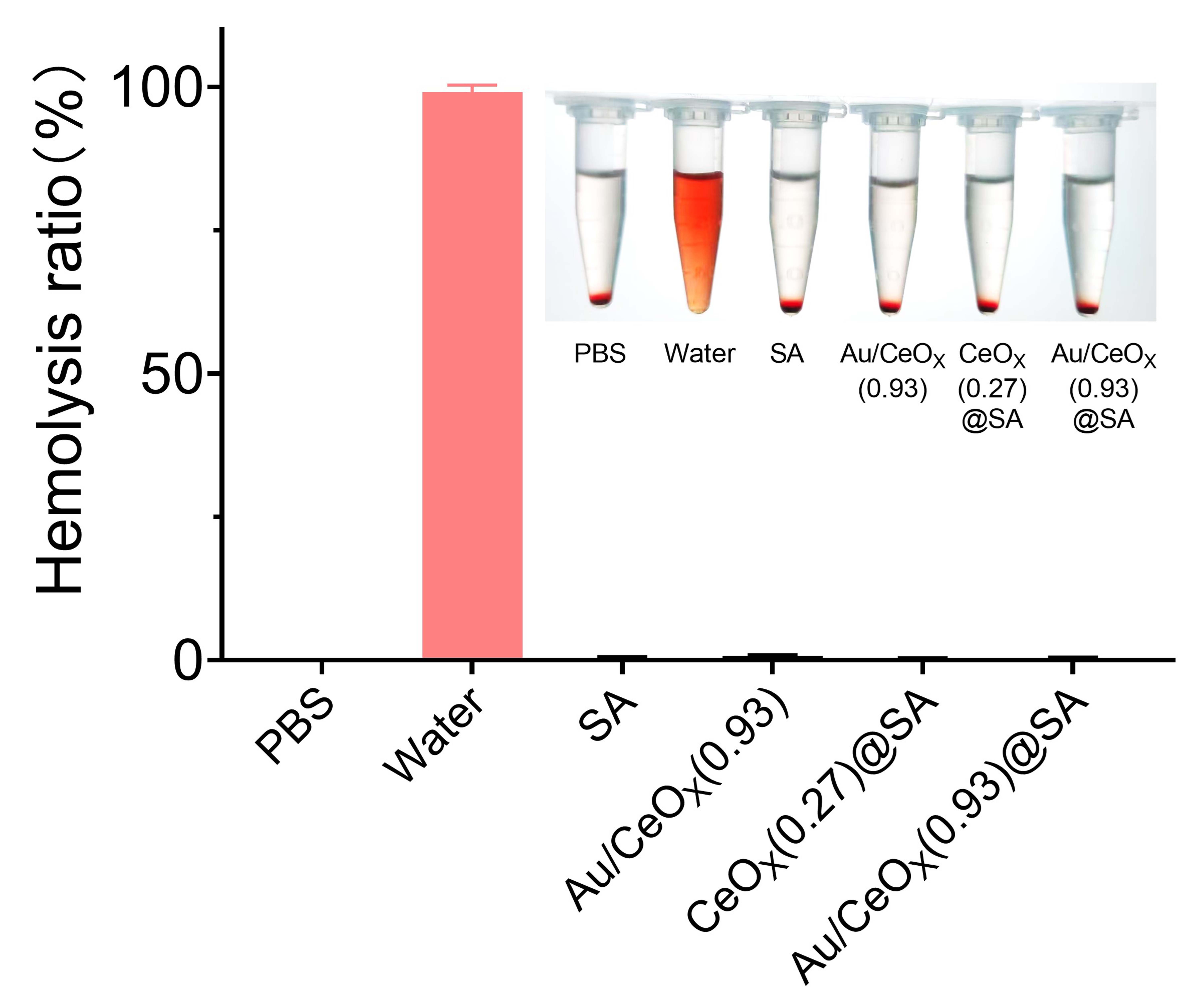


Figure S8 Hemolysis experiment of red blood cell incubated with Au/CeO_X_(0.93), CeO_X_(0.27)@SA, and Au/CeO_X_(0.93)@SA





Figure S9 Single-channel fluorescence images of LPS inducing RAW264.2 cells under different concentration treatments of CeO_X_(0.27) and Au/CeO_X_(0.93) for (A) detecting ROS levels when using DCFH-DA probe, for (B) detecting O_2_^-•^ levels when using DHE probe, for (C) assaying •OH when using HPF probe, for (D) detecting H_2_O_2_ levels when using ROSGreen^TM^ probe. All imaged cells are co-incubated with DAPI (blue).


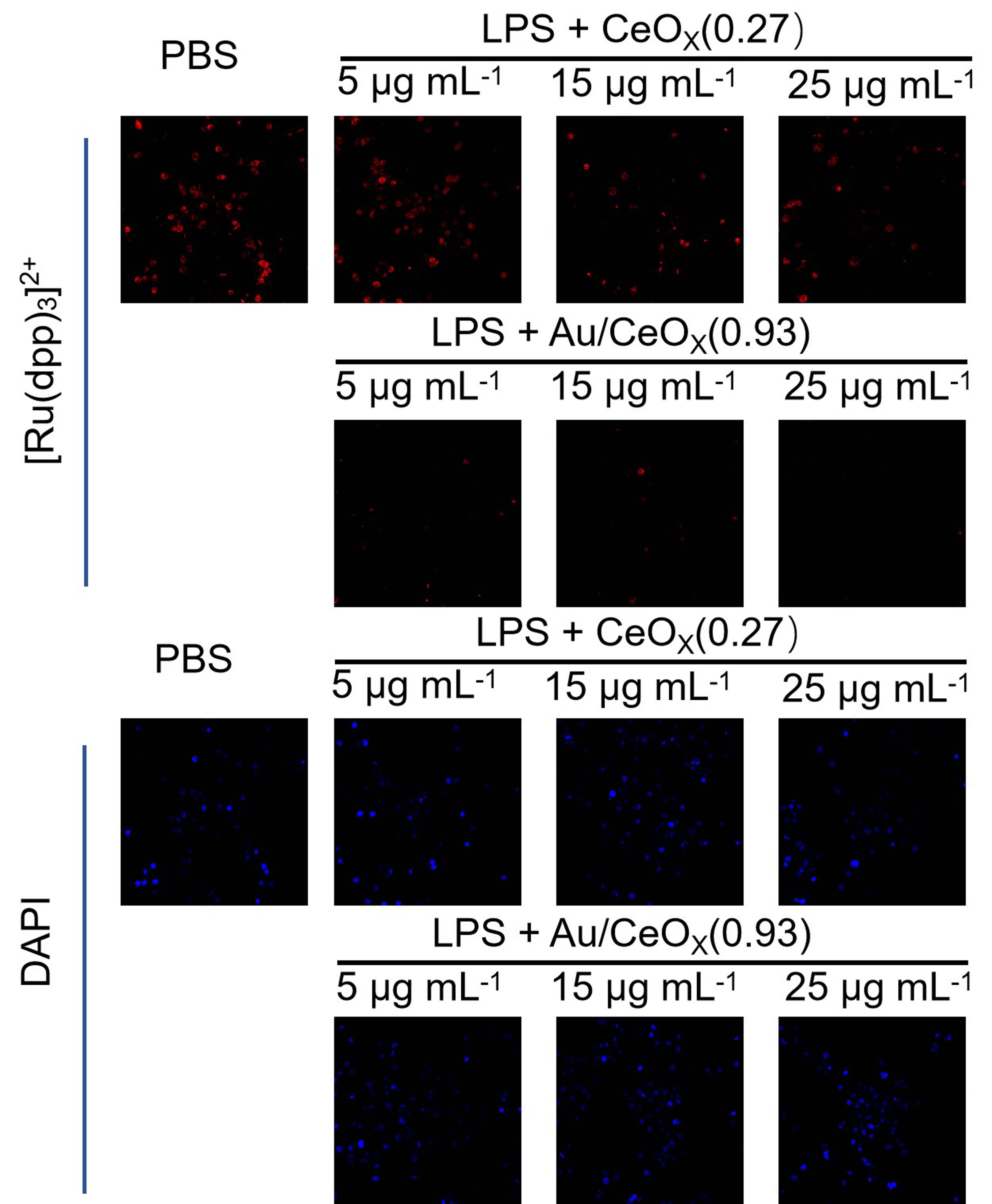


Figure S10 Single-channel fluorescence images of detecting O_2_ levels when using [Ru(dpp)_3_]^2+^ probe. All imaged cells are co-incubated with DAPI (blue).


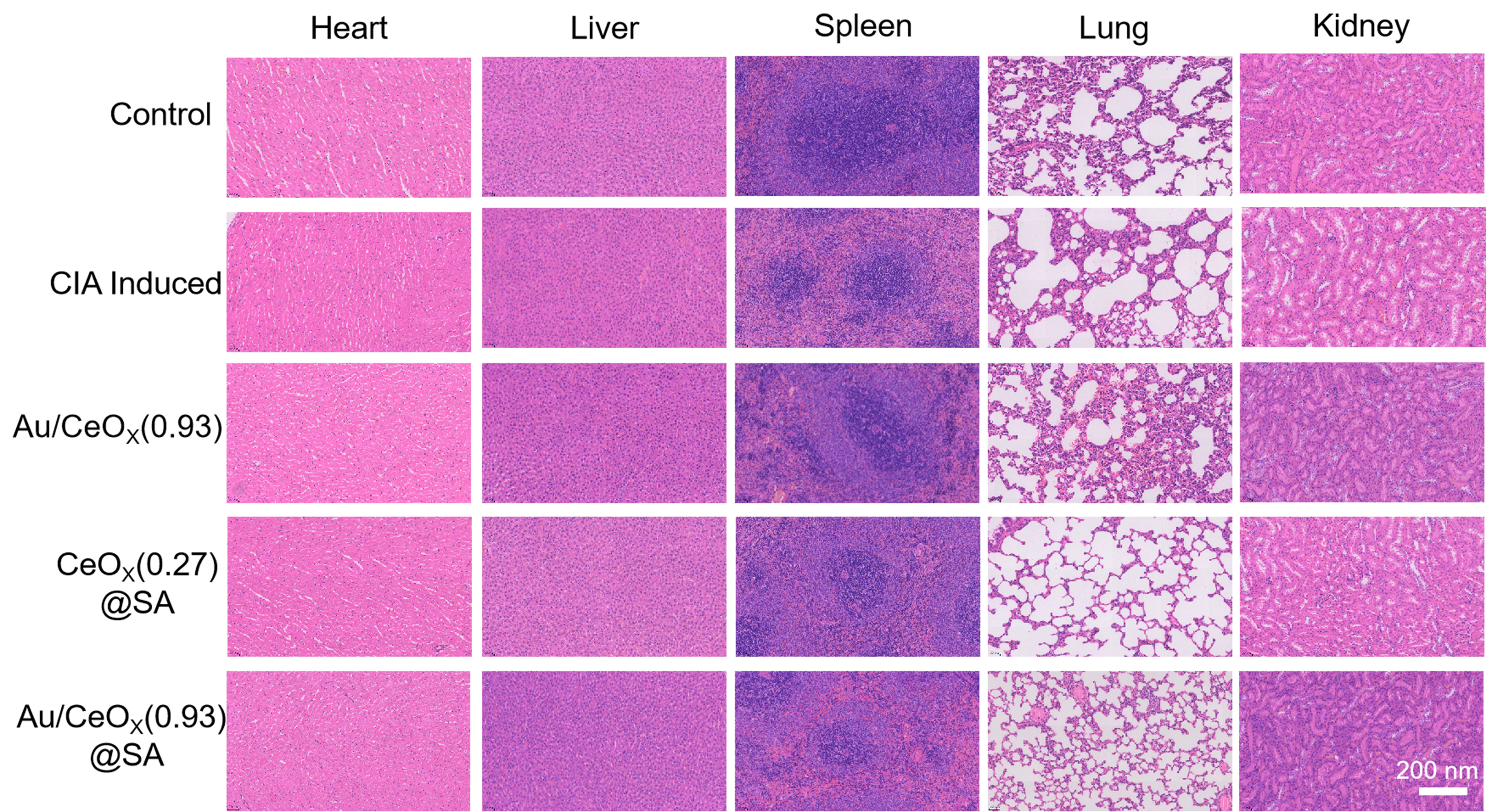


Figure S11 The H&E stained images of heart, liver, spleen, lungs, and kidneysin RA rats for assess the tissue biocompatibility of CeO_X_(0.27)@SA, CeO_X_(0.27)@SA and Au/CeO_X_(0.93)@SA nanoclusters.



Figure S12 α-diversity analysis of the gut microbiota at the genus level based on (A and B) Shannon, (C and D)Faith and (E and F)Evenness


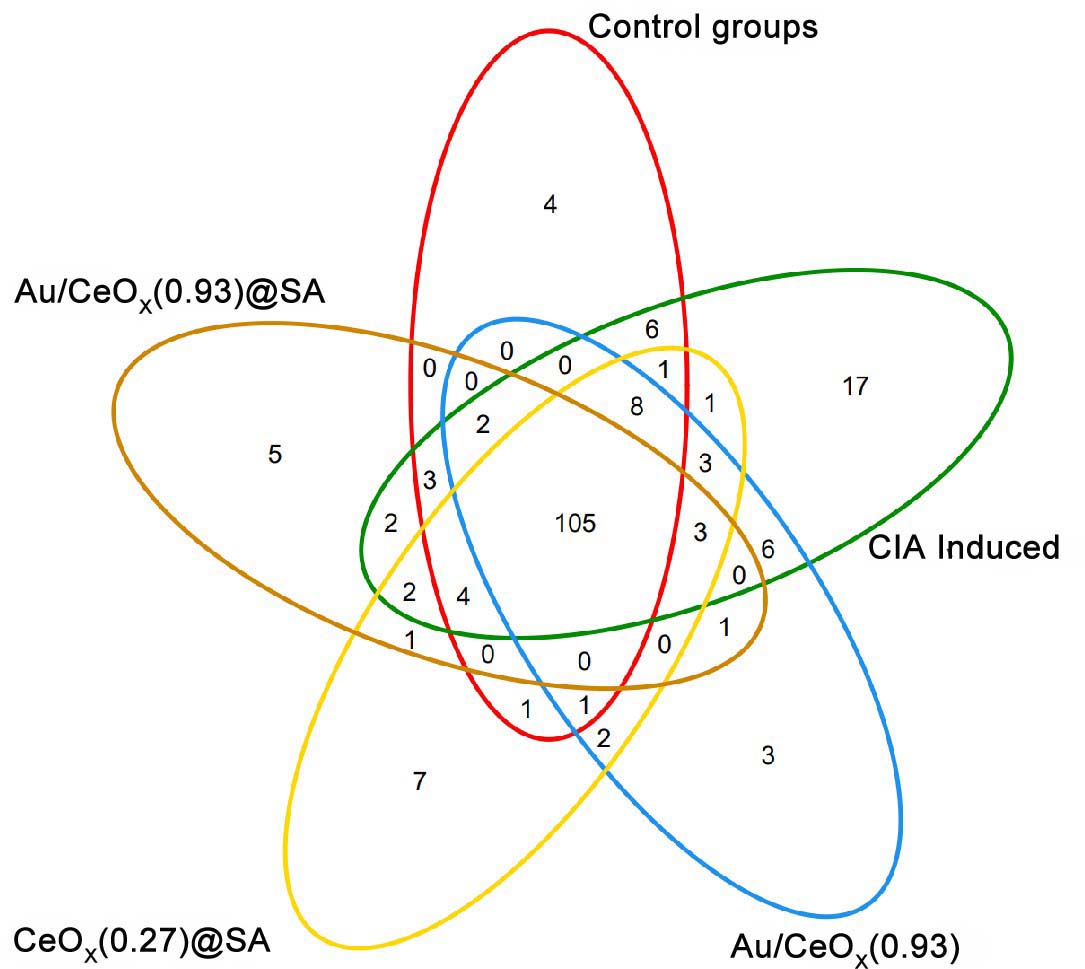


Figure S13 The genus-level Venn diagram analysis of the gut microbiota

Table S2 Scoring criteria for foot swelling in RA rats

| Score | Foot swelling |
| --- | --- |
| 0 | Normal, no obvious redness or swelling of the foot joints |
| 1 | Mild redness and swelling of the ankle and wrist joints |
| 2 | Moderate swelling of ankle, wrist joints |
| 3 | Severe redness and swelling of the paws, including the ends of the fingers |
| ≥4 | Severe redness and swelling at the ankle joint, with evidence of joint deformity or associated deformities |


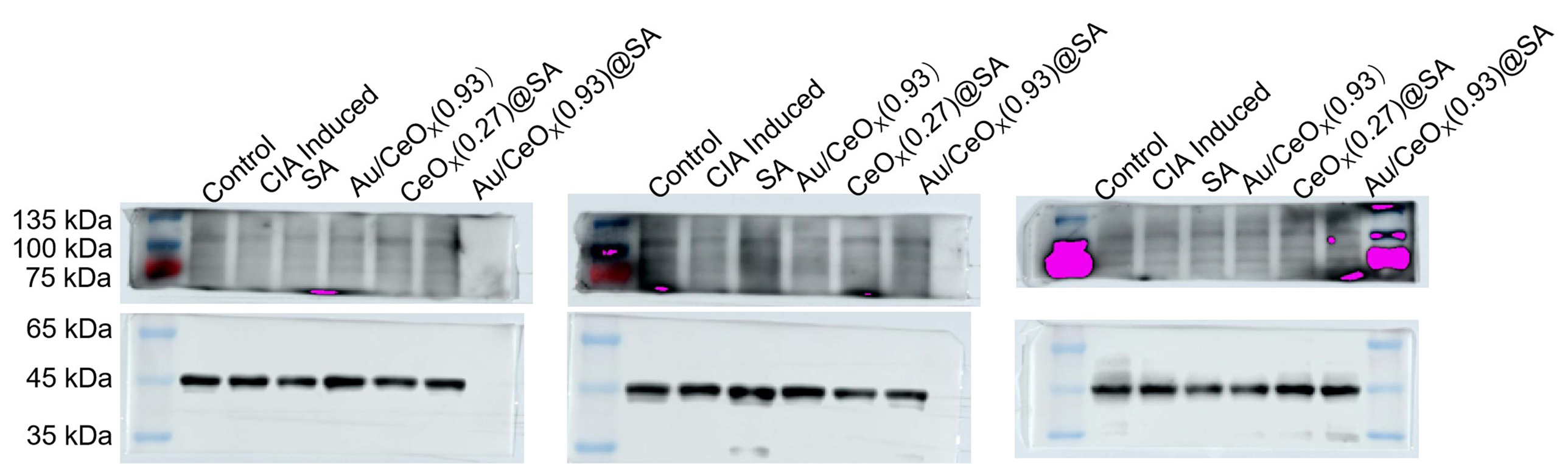


Figure S14 The expression levels of HIF-1α in colonic tissues were analyzed in original full-membrane Western Blot
